# Reproductive factors and lung cancer risk: a comprehensive systematic review and meta-analysis

**DOI:** 10.1186/s12889-020-09530-7

**Published:** 2020-09-25

**Authors:** Xin Yin, Zhiying Zhu, H. Dean Hosgood, Qing Lan, Wei Jie Seow

**Affiliations:** 1 Saw Swee Hock School of Public Health, National University of Singapore and National University Health System, Singapore, 117549 Singapore; 2 Division of Cancer Epidemiology and Genetics, National Cancer Institute, National Institutes of Health, Rockville, MD 20850 USA; 3 Dermatology Service, Department of Medicine, Memorial Sloan Kettering Cancer Center, New York, NY 10065 USA; 4 Department of Epidemiology and Population Health, Albert Einstein College of Medicine, The Bronx, NY 10461 USA; 5 Department of Medicine, Yong Loo Lin School of Medicine, National University of Singapore and National University Health System, Singapore, 119228 Singapore

**Keywords:** Lung cancer, Meta-analysis, Reproductive factors, Never-smokers, Parity

## Abstract

**Background:**

A number of studies have investigated the association between reproductive factors and lung cancer risk, however findings are inconsistent. This meta-analysis aimed to evaluate the association between female reproductive factors and lung cancer risk.

**Methods:**

We conducted a comprehensive systematic search to identify relevant and eligible studies published before 18th December 2019. Inter-study heterogeneity was assessed using the *Q* test and *I*^*2*^ statistic. Based on the heterogeneity of each reproductive factor, fixed or random effects models were used to calculate the summary odds ratios (ORs) and 95% confidence intervals (CIs). Subgroup analyses by study design, lung cancer subtypes, smoking status, and ethnicity were also performed.

**Results:**

A total of 66 studies with 20 distinct reproductive factors were included in this meta-analysis. Comparing the highest and lowest categories (reference) of each reproductive factor, parity (OR = 0.83, 95% CI = 0.72–0.96), menstrual cycle length (OR = 0.79, 95% CI = 0.65–0.96), and age at first birth (OR = 0.85, 95% CI = 0.74–0.98), were significantly associated with a lower risk of overall lung cancer. On the contrary, non-natural menopause was significantly associated with higher lung cancer risk (OR = 1.52, 95% CI = 1.25–1.86). Among never-smokers, a significant negative association was found between parity and lung cancer risk. Both parity and non-natural menopause were statistically significant in case-control studies.

**Conclusion:**

These results suggest that certain reproductive factors may be associated with lung cancer risk. Future studies should further validate the associations, and investigate the underlying mechanisms.

## Background

Lung cancer is the leading cause of cancer death worldwide among both men and women [1]. Despite cigarette smoking being the predominant carcinogen for lung cancer risk, it only contributes to approximately half of the lung cancer cases among women, since most women are never-smokers [2]. Among never smokers, the rate of lung cancer incidence was reported to be higher in women than in men [3], and a reduction in smoking prevalence decreased lung cancer mortality rate in men, but not in women [4]. Studies have suggested that women, by being more susceptible to carcinogens in tobacco smoke [5, 6], may have a higher chance of developing lung cancer at a younger age and with less smoking intensity than men [7–9].

Reproductive and hormonal factors have been hypothesized to be influential stimuli to lung cancer carcinogenesis. Previous studies have detected estrogen receptors in both normal and cancerous lung tissues [10, 11], higher rates of epidermal growth factor receptor (*EGFR*) mutation-positive lung cancer in never-smoking women [12], familial aggregation of reproductive cancers among female lung cancer patients [13, 14], and increased lung cancer risk in female cancer survivors with a history of reproductive-related primary tumors [15–21]. However, results from epidemiological studies investigating the relationship between hormonal and reproductive factors with lung cancer risk are conflicting. For example, some studies suggested a significant decrease of female lung cancer risk with increased parity [22–24] or hormone use [25–27], whereas other studies reported the opposite, a positive association between increased parity [28, 29] or hormone use [30, 31] and lung cancer risk.

To evaluate the association between female reproductive factors and lung cancer risk, we conducted a comprehensive systematic review and meta-analysis and stratified by ethnicity, smoking status, study design, and histology.

## Methods

### Literature search and identification of eligible studies

A systematic literature search was performed by two independent reviewers using the following search terms: reproductive, estrogen, hormone, birth, menopause, menarche, oral contraceptive, parity, pregnancy AND women AND lung cancer, using PubMed, Chinese National Knowledge Infrastructure (CNKI), National University of Singapore Library, and Google Scholar databases before December 2019, restricted to English and Chinese language papers. The detailed search strategy was developed for PubMed and adapted for other databases (Supplementary Table S1). Relevant publications from the reference lists of identified papers were also extensively reviewed to include additional studies in order to avoid missing any potential publications during the database search. Studies were scrutinized for their eligibility to be included in our analysis using the following inclusion criteria: 1) the study design was either case-control, cohort, or randomized controlled trial; 2) the outcome of interest was either overall lung cancer or lung cancer subtypes (adenocarcinoma (AC), squamous cell carcinoma (SCC), small cell lung cancer (SCLC) and non-small-cell lung cancer (NSCLC)); 3) the exposure variables were related to reproductive, menstrual, or hormonal factors; 4) if there were repeated studies published by the same group of authors, only the most recently updated publication was included, unless different study designs were used. Studies were excluded if they did not specify the reference group used, reference group overlapped, or if they did not include a measure of association.

### Data extraction

Information was extracted from each of the selected studies and recorded as the following variables: last name and initial of first name of the first author, publication year, journal name, reproductive factors, non-reference (the highest or the lowest) and reference group, study population (e.g., Asian, Caucasian, or Mixed), lung cancer subtype (AC, SCC, NSCLC or SCLC), study design (case-control, cohort, randomized clinical trial), number of cases and controls, smoking status (if applicable, never smokers, past smokers, current smokers), presence of *EGFR* mutation (if applicable), association estimates (odds ratios (ORs) or risk ratios (RRs) for case-control studies, risk ratios (RRs), standardized incidence ratios (SIRs) or hazard ratios (HRs) for cohort studies), 95% confidence intervals (CIs) and *P*-values for the non-reference group (for categorical variables) or for the continuous interval (for continuous variables), or P-trend with the corresponding statistical adjustments. As hormone replacement therapy type medications were reported in many different ways, including estrogen plus progestin (EPT), estrogen alone (E), hormone replacement therapy (HRT), hormone use, and postmenopausal hormone therapy, we combined them into an ever/never use of hormones variable; women with current and former smoking status were combined as “ever smokers” category. We recalculated the overall total effect using meta-analyses if the original paper only provided the separate effect for these single categories.

### Statistical analysis

Pooled estimates were calculated as the inverse variance-weighted mean of the logarithm of OR with 95% confidence interval (CI) to assess the association between reproductive factors and lung cancer risk. Heterogeneity among the included studies was evaluated using the *Q* test, and *I*^*2*^ statistic that represents the proportion of total variation attributable to inter-study heterogeneity. In the presence of substantial heterogeneity (*I*^*2*^ > 50%) [32], the random effects model was used as the pooling method; otherwise, the fixed effects model was applied. We also stratified the meta-analysis by study design (case-control, cohort or randomized clinical trial), lung cancer subtypes (overall lung cancer, AC or NSCLC), smoking status (never smokers or ever smokers), and ethnicity (Asian, Caucasian, or Mixed) for reproductive factors.

Assessment for potential publication bias was conducted using Egger’s linear regression analysis and trim-and-fill method. The ‘leave one out’ sensitivity analysis was carried out to assess potential heterogeneity and the robustness of the findings [33, 34].

To assess the quality of our included studies, we performed the Newcastle-Ottawa Scale (NOS) for observational studies and the Cochrane Risk of Bias Tool (CRBT) assessment for randomized controlled trials. For those studies with poor quality and high risk of bias (NOS score ≤ 5 or CRBT score < 4), we excluded them for the sensitivity analysis.

This meta-analysis followed the Preferred Reporting Items for Systematic Reviews and Meta-Analyses (PRISMA) guidelines [35]. All statistical analysis was conducted using Stata version 15.0 (Stata Corporation, College Station, Texas, USA). All statistical tests were conducted as two-sided, and a *P*-value of < 0.05 was considered as being statistically significant.

## Results

**Fig. 1:**
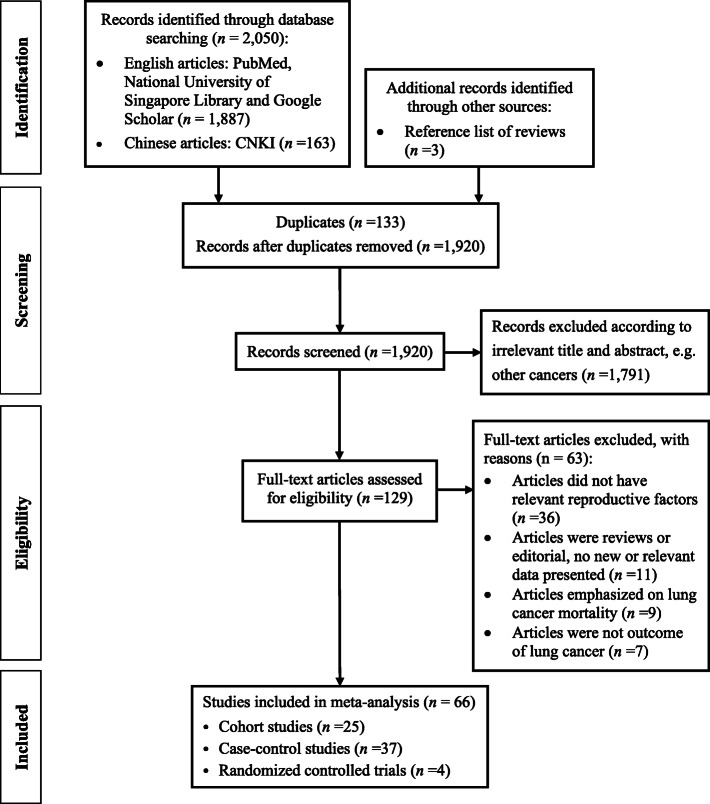
Flowchart of study selection, inclusion, and analysis

**Fig. 2:**
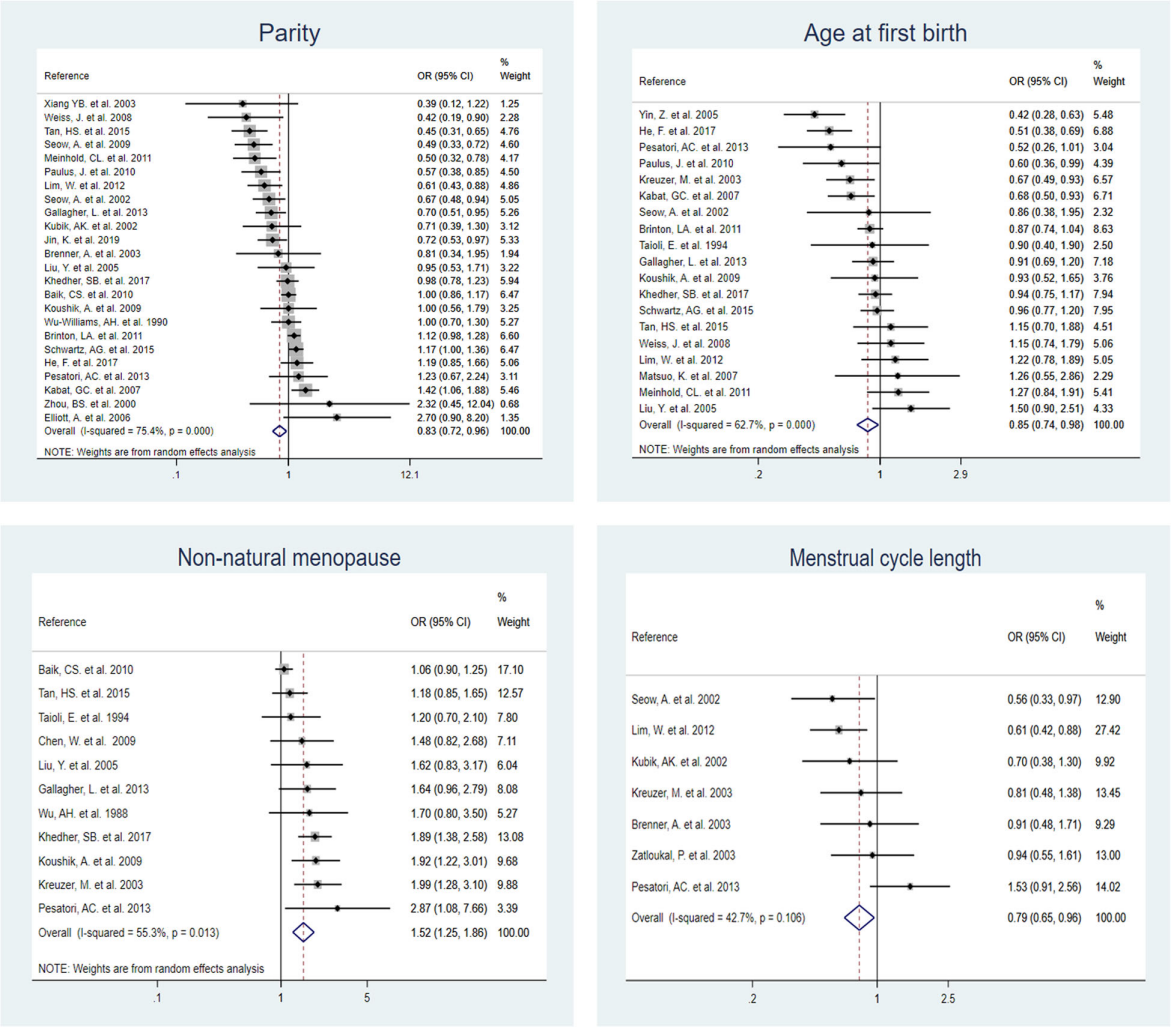
Significant associations between reproductive variables and overall lung cancer risk

The literature search identified 2050 publications from the databases and 3 additional studies were retrieved from the reference lists of previous meta-analysis studies that were identified through the search terms. A total of 133 duplicates, 1791 irrelevant publications, and 63 studies with full-text screening were excluded. Finally, 66 studies were eligible for inclusion into this meta-analysis [22–28, 31, 36–93] (Fig. 1). The characteristics of the 66 selected studies are shown in Supplementary Table S2, Additional file 1. In total, there were 25 cohort studies, 37 case-control studies, and four randomized controlled trials. The publication years of these studies ranged from 1987 to 2019. Collectively, 26 studies were conducted among Asian females, 25 studies among Caucasian females, and 15 studies among mixed ethnicities. Of the 66 selected studies, 20 studies further stratified by smoking status, and 23 studies stratified by lung cancer subtypes (AC, SCC, NSCLC or SCLC). A total of 31 reproductive factors were extracted from the selected studies, with 20 reproductive factors included in the meta-analysis after combining some of the factors due to the sparse number of eligible studies (Table 1 and Fig. 1). Among these exposures, we found statistical significance for four reproductive variables with overall lung cancer risk: parity, age at first birth, non-natural menopause, and menstrual cycle length (Fig. 2). Other forest plots are shown in Supplementary Fig. S1, Additional file 2.

**Table 1:** Association of reproductive factors and overall lung cancer risk (highest vs. lowest category)

Reproductive factors	Highest category	Lowest category (reference)	No. of studies	*P*_het_^a^	*I*^*2*^ value (%)	OR (95% CI)^b^
Menstrual-related factors						
Age at menopause	≥50 to ≥55	Premenopausal or < 50	22	< 0.001	64.3	0.99 (0.88, 1.12)
Age at menarche	≥15 to ≥18	< 11 to ≤15	20	0.032	40.4	1.03 (0.96, 1.10)
**Non-natural menopause**	**Non-natural**	**Natural / premenopausal**	**11**	**0.013**	**55.3**	**1.52 (1.25, 1.86)**
**Ovariectomy**	**yes**	**no**	**5**	**0.487**	**0.0**	**1.38 (1.16, 1.64)**
Hysterectomy	yes	no	4	0.171	40.1	1.21 (0.98, 1.49)
Ovariectomy and Hysterectomy	yes	no	4	0.405	0.0	1.22 (0.95, 1.58)
**Menstrual cycle length**	**> 30 days**	**< 27 to ≤ 30 days**	**7**	**0.106**	**42.7**	**0.79 (0.65, 0.96)**
Menopausal status	Post-menopausal	Pre-menopausal	6	0.057	53.4	1.26 (0.92, 1.73)
Length of menstrual flow (days)	≥5 to > 6	≤3 to < 5	4	0.712	0.0	1.01 (0.84, 1.23)
Other factors						
Hormone use	ever	never	38	< 0.001	56.6	0.95 (0.90, 1.01)
Oral contraceptive use	ever	never	26	0.003	48.2	1.01 (0.94, 1.09)
OC use duration (years)	≥2 to ≥12	0 to < 2	12	0.012	54.5	0.99 (0.87, 1.14)
**Parity**	**≥3 to ≥ 7**	**0 to 2**	**24**	**< 0.001**	**75.4**	**0.83 (0.72, 0.96)**
Number of pregnancy	≥ 4 to ≥7	0 to 2	10	0.002	66.3	0.91 (0.73, 1.15)
**Age at first birth**	**≥25 to ≥ 31**	**Nulliparous or < 25**	**19**	**< 0.001**	**62.7**	**0.85 (0.74, 0.98)**
Reproductive period (years)	≥36 to ≥41	≤30 to < 33	8	0.048	50.6	0.95 (0.78, 1.17)
Breastfeeding	ever	never	6	0.330	13.2	0.94 (0.83, 1.06)
Miscarriage	ever	never	4	0.063	58.9	1.20 (0.93, 1.56)
Tubal sterilization use	ever	never	4	0.019	69.7	1.05 (0.84, 1.33)
Intrauterine device use	ever	never	4	0.097	52.5	0.83 (0.66, 1.04)

^a^Heterogeneity *P*-value

^b^Adjusted odds ratio (OR) and 95% confidence interval (CI). Highest non-reference category as compared to the lowest reference group

### Parity

Twenty-four studies reported the association between parity and overall lung cancer risk. A total of 10 studies stratified by lung cancer subtypes. The highest parity category ranged from ≥3 to ≥7 children, while the lowest parity category ranged from 0 to 2 children. The pooled ORs were 0.83 (95% CI = 0.72–0.96, *I*^*2*^ = 75.4%) for overall lung cancer, 0.84 (95% CI = 0.63–1.11, *I*^*2*^ = 75.1%) for NSCLC, and 0.83 (95% CI = 0.63–1.09, *I*^*2*^ = 67.0%) for adenocarcinoma (Tables 1 and 2). Specifically, a significant negative association of higher parity and overall lung cancer risk was found among Asians (OR = 0.70, 95% CI = 0.58–0.86, *I*^*2*^ = 59.7%), but no association was observed among Caucasians. After further stratification by study design, and lung cancer subtype, higher parity was significantly associated with decreased risks of overall lung cancer (OR = 0.58, 95% CI = 0.44–0.75, *I*^*2*^ = 45.3%), non-small cell lung cancer (OR = 0.39, 95% CI = 0.18–0.84, *I*^*2*^ = 25.3%) and lung adenocarcinoma (OR = 0.45, 95% CI = 0. 28–0.71, *I*^*2*^ = 30.0%) among Asian women in cohort studies.

**Table 2:** Association of significant reproductive factors and lung cancer risk, stratified by subgroups (highest vs. lowest category)

Reproductive factors^a^	n	OR (95% CI)^b^	Case-Control	Cohort		
			n	OR (95% CI)^b^	n	OR (95% CI)^b^
Parity (highest vs. lowest)						
Overall lung cancer	24	**0.83 (0.72, 0.96)**	15	**0.82 (0.68, 0.98)**	9	0.85 (0.68, 1.05)
Asian	12	**0.70 (0.58, 0.86)**	7	0.81 (0.65, 1.01)	5	**0.58 (0.44, 0.75)**
Caucasian	8	1.03 (0.85, 1.25)	5	0.93 (0.60, 1.43)	3	1.12 (0.96, 1.31)
Adenocarcinoma	10	0.83 (0.63, 1.09)	5	1.00 (0.82, 1.22)	5	0.71 (0.43, 1.16)
Asian	5	0.72 (0.37, 1.39)	2	1.22 (0.86, 1.73)	3	**0.45 (0.28, 0.71)**
Caucasian	4	0.93 (0.76, 1.13)	2	0.74 (0.44, 1.24)	2	0.97 (0.78, 1.20)
Non-small cell lung cancer	10	0.84 (0.63, 1.11)	5	0.81 (0.51, 1.29)	5	0.85 (0.57, 1.27)
Asian	4	0.82 (0.33, 2.07)	2	1.22 (0.86, 1.73)	2	**0.39 (0.18, 0.84)**
Caucasian	4	0.94 (0.69, 1.29)	2	0.74 (0.44, 1.24)	2	0.97 (0.78, 1.20)
Never Smokers	11	**0.71 (0.54, 0.93)**	5	**0.65 (0.52, 0.81)**	6	0.80 (0.51, 1.28)
Asian	7	**0.65 (0.54, 0.78)**				
Caucasian	2	0.99 (0.25, 3.85)				
Ever Smokers	8	0.78 (0.59, 1.04)	4	**0.56 (0.41, 0.75)**	4	1.01 (0.76, 1.34)
Asian	4	**0.54 (0.40, 0.75)**				
Caucasian	2	1.14 (0.92, 1.40)				
Parity (highest vs. nulliparous)						
Overall lung cancer	16	**0.77 (0.62, 0.95)**	10	0.79 (0.62, 1.00)	6	0.72 (0.49,1.06)
Asian	5	**0.56 (0.47, 0.67)**	1	**0.61 (0.43, 0.87)**	4	**0.55 (0.45, 0.66)**
Caucasian	8	0.96 (0.73, 1.26)	6	0.83 (0.58, 1.18)	2	1.22 (0.97, 1.52)
Adenocarcinoma	8	0.71 (0.49, 1.01)	4	0.83 (0.64, 1.08)	4	0.66 (0.31, 1.41)
Asian	3	**0.45 (0.28, 0.71)**			3	**0.45 (0.28, 0.71)**
Caucasian	4	0.84 (0.52, 1.35)	3	0.66 (0.45, 0.98)	1	1.41 (0.90, 2.20)
Age at first birth						
Overall lung cancer	19	**0.85 (0.74, 0.98)**	12	**0.77 (0.61, 0.97)**	7	0.94 (0.81, 1.08)
Asian	9	0.90 (0.66, 1.22)				
Caucasian	6	**0.75 (0.65, 0.88)**				
Adenocarcinoma	10	**0.84 (0.74, 0.95)**	6	0.85 (0.72, 1.00)	4	0.82 (0.67, 1.01)
Asian	3	**0.62 (0.47, 0.83)**				
Caucasian	5	0.82 (0.68, 0.97)				
Non-natural menopause						
Overall lung cancer	11	**1.52 (1.25, 1.86)**	6	**1.83 (1.50, 2.22)**	5	**1.17 (1.00, 1.37)**
Asian	4	**1.36 (1.07, 1.72)**				
Caucasian	5	**1.67 (1.12, 2.49)**				
Adenocarcinoma	6	**1.41 (1.18, 1.69)**	5	**1.64 (1.25, 2.14)**	1	1.24 (0.97, 1.59)
Caucasian	5	**1.44 (1.19, 1.74)**				
Menstrual cycle length						
Overall lung cancer	7	**0.79 (0.65, 0.96)**	7	**0.79 (0.65, 0.96)**		
Asian	3	**0.64 (0.49, 0.85)**				
Caucasian	4	0.98 (0.74, 1.28)				
Adenocarcinoma	4	0.79 (0.55, 1.14)	4	0.79 (0.55, 1.14)		
Caucasian	3	0.79 (0.51, 1.21)				

^a^Subgroup analyses were conducted if there were at least four studies published for that reproductive variable

^b^Adjusted odds ratio (OR) and 95% confidence interval (CI). Highest non-reference category as compared to the lowest reference group

Eleven studies assessed parity and lung cancer risk among never-smokers while 8 studies assessed the association among ever-smokers. For overall lung cancer, the ORs for Asian women were 0.65 (95% CI = 0.54–0.78, *I*^*2*^ = 0.0%) among never-smokers and 0.54 (95% CI = 0.40–0.75, *I*^*2*^ = 0.0%) among ever smokers. Among case-control studies, higher parity was also significantly negatively associated with overall lung cancer risk among never smokers (OR = 0.65, 95% CI = 0.52–0.81, *I*^*2*^ = 0.0%).

Relative risk estimates of ever parous (≥1 child versus nulliparous) were reported in 16 lung cancer studies and 8 adenocarcinoma studies. We also found a significant negative association between parity and overall lung cancer risks among Asian women in both case-control and cohort studies (OR = 0.56, 95% CI = 0.47–0.67, *I*^*2*^ = 11.4%). Forest plots of subgroup analyses are shown in Supplementary Figs. S2-S8, Additional file 2.

### Age at first birth

Relative risk estimates for age at first birth were reported in 19 studies for overall lung cancer and 10 studies for adenocarcinoma (oldest age group versus youngest age group). The highest age at first birth category ranged from ≥25 to ≥31, and the lowest reference age at first birth category ranged from nulliparous to < 25. The pooled ORs were 0.85 (95% CI = 0.74–0.98, *I*^*2*^ = 62.7%) and 0.84 (95% CI = 0.74–0.95, *I*^*2*^ = 36.9%) for overall lung cancer and adenocarcinoma risk for older age at first birth, respectively (Tables 1 and 2). Among Asian women, older age at first birth was significantly negatively associated with adenocarcinoma risks (OR = 0.62, 95% CI = 0.47–0.83, *I*^*2*^ = 0.0%). Among Caucasian women with older age at first birth, overall lung cancer risk (OR = 0.75, 95% CI = 0.65–0.88, *I*^*2*^ = 15.9%) was significantly lower than those with younger age at first birth, but this association was not significant among Asian women. We did a sensitivity analysis by excluding the nulliparous women to avoid conflating the effects of parity and age at first birth (only one study was excluded), and the result did not change (OR = 0.83, 95% CI = 0.72–0.96, *I*^*2*^ = 61.7%). Forest plots of subgroup analyses are shown in Supplementary Fig. S9, Additional file 2.

### Non-natural menopause

Relative risk estimates for non-natural menopause versus natural menopause were reported in 11 studies for overall lung cancer (OR = 1.52, 95% CI =1.25–1.86, *I*^*2*^ = 55.3%) and in 6 studies for adenocarcinoma (OR = 1.41, 95% CI =1.18–1.69, *I*^*2*^ = 0.0%) (Tables 1 and 2). Non-natural menopause was significantly associated with higher overall lung cancer risk, particularly among Caucasian women for overall lung cancer (OR = 1.67, 95% CI =1.12–2.49, *I*^*2*^ = 73.4%) and lung adenocarcinoma (OR = 1.44, 95% CI =1.19–1.74, *I*^*2*^ = 0.0%). A significant positive association with overall lung cancer risk was found among women with ovariectomy (OR = 1.38, 95% CI =1.16–1.64, *I*^*2*^ = 0.0%) (Supplementary Fig. S1, S10, Additional file 2).

### Menstrual cycle length

The highest menstrual cycle length category was defined as more than 30 days, and the lowest reference menstrual cycle length category ranged from < 27 to ≤30 days. The association between menstrual cycle length (highest vs. lowest category) and lung cancer risk was reported in 7 studies for overall lung cancer (OR = 0.79, 95% CI = 0.65–0.96, *I*^*2*^ = 42.7%) and in 4 studies for adenocarcinoma (OR = 0.79, 95% CI = 0.55–1.14, *I*^*2*^ = 0.0%) (Tables 1 and 2). Longer menstrual length was significantly associated with lower lung cancer risks among Asian women (OR = 0.64, 95% CI = 0.49–0.85, *I*^*2*^ = 0.0%) (Supplementary Fig. S11, Additional file 2).

### Publication bias

Publication bias was assessed using Egger’s test, if there were at least 10 studies on the reproductive factor [94]. Results of Egger’s test showed that studies among parity and menopause type may have publication bias (Table 3). This suggested the presence of a potential publication bias, a language bias, inflated estimates by a flawed methodologic design in smaller studies, and/or a lack of publication of small trials with opposite results. Therefore, we conducted the ‘leave one out’ sensitivity analysis to explore the heterogeneity among studies of parity and menopause type, and no individual study was found to have excessive influence on the pooled effect (Supplementary Fig. S12, Additional file 2).

**Table 3:** Egger’s test for publication bias assessment (number of studies ≥10)

Variables	No. of studies	*P* value of Egger’s test
Hormone use	38	0.498
OC use	26	0.565
**Parity**	24	**0.041**
Age at menopause	22	0.984
Age at menarche	20	0.841
Age at first birth	19	0.895
Parity with nulliparous women as reference	16	0.110
OC use duration	12	0.112
**Menopause type**	11	**0.014**
Number of pregnancy	10	0.559

### Sensitivity analysis

In addition, we used the trim and fill method to test publication bias that conservatively imputes hypothetical negative unpublished studies to mirror the positive studies that cause funnel plot asymmetry. The imputed studies produce a symmetrical funnel plot (Supplementary Fig. S13, Additional file 2). The pooled analysis incorporating the hypothetical studies continued to show a statistically significant association between parity, menopause type, and lung cancer risk.

We assessed the quality of all the included studies by removing 7 studies with poor quality and a high level of bias (NOS score ≤ 5 or CRBT score < 4) for the sensitivity analysis (Supplementary Table 3–9, Additional file 1). The results did not change except for hormone use, and the effect of hormone use changed from borderline non-significant (OR = 0.95, 95% CI = 0.90–1.01, *I*^*2*^ = 56.6%) to borderline significant (OR = 0.93, 95% CI = 0.88–0.99, *I*^*2*^ = 55.4%).

## Discussion

Our meta-analysis of 20 reproductive factors revealed significant associations between parity, non-natural menopause, menstrual cycle length, and age at first pregnancy on lung cancer risk. Higher parity, older age at first pregnancy, and longer menstrual cycle length were associated with lower lung cancer risk. Conversely, non-natural menopause such as ovariectomy was found to be associated with higher risk of lung cancer.

Previous meta-analysis studies reported no significant associations between parity and lung cancer risk, regardless of ethnicity or study design [95, 96]. However, the number of included studies and different methods of calculating the risk ratios may account for the discrepancy. Two previous meta-analysis studies included a total of up to 21 studies before 2012 [95, 96]. They combined the estimates of the number of pregnancy and live birth, and Dahabreh et al.’s meta-analysis study [95] used both continuous and categorical risk estimates from published studies. In contrast, for parity, our meta-analysis only used categorical relative risk estimates extracted from 24 published studies and included 8 recent new studies after 2012 in addition to all the other studies that were included in the previous two meta-analyses. Relative risk estimates for the number of pregnancies and overall lung cancer risk reported by eight studies were included as an independent variable in our meta-analysis. Similar to Zhang et al. [96], we also found a significant negative association of lung cancer risk among women with longer menstrual cycle length. Shorter menstrual cycle length may increase the period of endogenous estrogen exposure (follicular phase), followed by increased cumulative exposure [97]. However, a previous meta-analysis found no significant association between older age at first birth and lung cancer risk [96].

There are several proposed mechanisms that have been hypothesized to explain the relationship between reproductive factors and the risk of lung cancer. Collectively, these factors are ascribed to the potential effects of estrogen on lung cancer risk. The hormonal etiology may play a direct role in the development of lung cancer. Estrogen and progesterone were associated with lung tumor proliferation, a process that can be triggered by hormonal receptors including estrogen receptors (ERs), progesterone, and epidermal growth factor (EGFR) receptors [98]. These receptors were found to be expressed in lung tumors [99, 100] and normal lung tissues [101], and they demonstrated regulatory effects in tumor growth and proliferation [10, 102–104]. Progesterone- receptors were reported to have tumor-suppressive effects, [105] while estrogen receptors were shown to stimulate tumor proliferation [106, 107]. Estrogen levels among women with lung cancer are usually higher than those in women without lung cancer [108]. Estrogenic stimulation in a murine xenograft model produced proliferative responses in lung tumor cell lines and increased tumor volumes [11]. Previous studies also reported that estrogen β receptors promote estrogen-dependent growth of lung cancer cells [109, 110]. In our study, we found negative associations between higher parity, older age at first pregnancy, and longer menstrual cycle length with lung cancer risk. Consistent with the proposed mechanisms, these protective reproductive factors are associated with lower estrogen levels in women [97, 111, 112].

Furthermore, estrogen can directly stimulate the transcription of estrogen-responsive genes in the nucleus of lung cells, and transactivate growth factor signaling pathways, in particular the epidermal growth factor pathway [100, 113]. *EGFR* mutations often occur among adenocarcinoma lung cancer subtypes, females, never-smokers, and East Asians [114–117]. In our study, we observed lower lung cancer risk among never-smokers and Asian women with higher parity, suggesting that higher parity is inversely associated with lung cancer risk by inhibiting *EGFR* activation or mutation. Estrogens may also influence lung carcinogenesis by their effect on carcinogen metabolism via the cytochrome P450 enzyme system [100].

We also found that non-natural menopause, including ovariectomy, was positively associated with lung cancer risk. However, the potential mechanisms remain unclear. This may be due to a sudden drop in circulating hormone levels after bilateral ovariectomy, unlike natural menopausal women whose circulating hormone levels decline gradually [65, 118, 119]. In addition, women with surgical menopause are usually placed on long-term hormone replacement therapy, which was previously shown to be associated with lung cancer risk [120]. However, there are controversies in the association between hormone replacement therapy and lung cancer risk [113].

Our meta-analysis has several limitations. First, we were unable to assess the dose-response effect or further stratify by other, or less common lung cancer histology types for most reproductive factors (e.g., squamous cell carcinoma, small cell lung cancer) because there were inadequate number of studies for such analyses. Second, the existing studies used different terms to describe hormone use (e.g., hormone replacement therapy, hormone therapy, postmenopausal hormone use, estrogen use, conjugated estrogen use, estrogen replacement use), thus we combined them and examined the overall association of hormone use on lung cancer risk. Hence, we may not be able to delineate the differential associations of the different hormone types. Third, given the different cutoffs used for reference category and confounder adjustments across different studies, our results might include trivial disparity and instability when evaluating the true impact of reproductive factors on lung cancer risk. Fourth, this analysis can only draw an inference on association of the reproductive factors with lung cancer risk, and not the cause-effect relationship. Finally, our inclusion of only published articles that were written either in English or in Chinese, and the exclusion of potentially relevant papers that were not publicly available may influence the publication bias of our study [121, 122].

Despite some of the existing limitations in our study, this is the first study to include a comprehensive review and meta-analysis of 20 reproductive factors, provide detailed stratification on each of the reproductive factor by lung cancer subtype, smoking status, ethnicity and study design, and assess the association between certain reproductive factors such as breastfeeding, ovariectomy, miscarriage, tubal sterilization use, reproductive period, length of menstrual flow, hysterectomy, intrauterine device use, and lung cancer risks among women.

## Conclusions

In conclusion, we found a significant protective effect of higher parity, older age at first pregnancy, and longer menstrual cycle length on lung cancer risk, but a significant positive association between non-natural menopause with lung cancer risk. Increased parity had a negative association with lung cancer risk among never-smoking women. Future studies should validate the association between reproductive and menstrual exposures on lung cancer risks and investigate the underlying mechanisms.

## Supplementary information

**Additional file 1: Supplementary Table S1.** Search strategy for relevant studies in PubMed. **Supplementary Table S2.** Characteristics of selected studies reporting the associations between reproductive factors and overall lung cancer risks. **Supplementary Table S3–8.** Quality assessment of the included studies. **Supplementary Table S9.** Sensitive analysis of the association of reproductive factors and overall lung cancer risk

**Additional file 2: Supplementary Figs. S1–13.** Forest plots for lung cancer risks in women with reproductive variables

## Data Availability

Not applicable.

## Abbreviations

OR: Odds ratio
CI: Confidence interval
EGFR: Epidermal growth factor receptor
CNKI: Chinese National Knowledge Infrastructure
AC: Adenocarcinoma
SCC: Squamous cell carcinoma
SCLC: Small-cell lung cancer
NSCLC: Non-small-cell lung cancer
RR: Risk ratio
SIR: Standardized incidence ratio
HR: Hazard ratio
EPT: Estrogen plus progestin
E: Estrogen alone
HRT: Hormone replacement therapy
ER: Estrogen receptor
NOS: Newcastle-Ottawa Scale
CRBT: Cochrane Risk of Bias Tool
PRISMA: Preferred Reporting Items for Systematic Reviews and Meta-Analyses
