# Supplementary material for: Reproductive factors and lung cancer risk: a comprehensive systematic review and meta-analysis
Source: BMC Public Health. 2020 Sep 25;20:1458. doi: 10.1186/s12889-020-09530-7 (PMC7519481; doi:10.1186/s12889-020-09530-7)
Supplement: Supplementary file 1 — Additional file 1: Supplementary Table S1. Search strategy for relevant studies in PubMed. Supplementary Table S2. Characteristics of selected studies reporting the associations between reproductive factors and overall lung cancer risks. Supplementary Table S3–8. Quality assessment of the included studies. Supplementary Table S9. Sensitive analysis of the association of reproductive factors and overall lung cancer risk [file 12889_2020_9530_MOESM1_ESM.docx]

**Supplementary Table S1. Search strategy for relevant studies in PubMed**

Search Terms 1: #1

((((((((reproductive) OR (estrogen)) OR (hormone)) OR (birth)) OR (menopause)) OR (menarche)) OR (oral contraceptive)) OR (parity)) OR (pregnancy)

Search Terms 2: #2

(women) OR (female)

Search Terms 3: #3

(lung) OR (pulmonary)

Search Terms 4: #4

((((((((cancer) OR (tumor)) OR (carcinoma)) OR (adenocarcinoma)) OR (neoplasm)) OR (squamous cell)) OR (NSCLC)) OR (SCLC)) OR (large cell carcinoma))

Search Terms 5: #5

#1 AND #2 AND #3 AND #4

**Supplementary Table S2. Characteristics of selected studies reporting the associations between reproductive factors and overall lung cancer risks**

| Authors, year (reference) | Region, Ethnicity | Country | Exposure(s) | Study design | Cases/control  Or  Case/total population^*^ (person year) | Comparison group | Age range or mean age (yrs.) | Histologic type of cases | Analyzed Histologic type | Smoking status | Analyzed Smoking group | Confounders |
| --- | --- | --- | --- | --- | --- | --- | --- | --- | --- | --- | --- | --- |
| Vohra, SN. et al. 2019 (1) | Asian | Nepal | Birth control use | Case-control study | 268/226 | Hospital-based case-controls | Cases 59.2 (±11.6) Controls 53.2 (±9.6) | Overall lung cancer | Overall lung cancer | Never smoker,  Ever smoker | - | all reproductive and hormonal exposures, in addition to adjustment for age and cumulative active smoking years; |
| Jin, K. et al. 2019(2) | Asian | China | Age at menarche, Age at menopause, Reproductive window, Parity, Gravidity, Number of live birth, Oral Contraceptive use | Case-control study | 680/1808 | Hospital-based case-controls | Cases 66.79 (±9.36) Controls 67.37 (±9.32) | Overall lung cancer | Overall lung cancer | Never smoker,  Ever smoker | Never smoker,  Ever smoker | age (as a continuous variable), smoking status (ever or never), pack-years of smoking, family history of lung cancer (yes or no), income, education, county of residence,  and BMI.  2. Odds ratios and |
| Titan, AL. et al. 2019(3) | Caucasian | USA | Pregnancy, Birth Control, HRT | Cohort study | 1147/75587 | Participants in Prostate, Lung, Colorectal, and Ovarian Cancer Screening Trial | 62.5 (±5.4) | Non-small cell lung cancer | Non-small cell lung cancer | Never smoker,  Former smoker,  Current smoker | - | age, smoking history, race, BMI, education, HRT use, comorbidity conditions of emphysema and bronchitis, family history of lung cancer, pregnancy number, birth control use, conditions of stroke, and cardiac disease |
| Iversen, L. et al. 2017 (4) | Caucasian | UK | oral contraceptives | Cohort study | 162/46022 | Participants in Royal College of General Practitioners’ Oral  Contraception Study | - | - | Overall lung cancer | Never smoker,  Ever smoker | Never smoker,  Ever smoker | Poisson regression adjusted for age, parity, smoking, and social status |
| He, F. et al. 2017 (5) | Asian | China | Age at menarche, Number of live births,  Age at first birth,  Age at menopause,  Breastfeeding,  Contraceptive history (Contraceptive usage and hormone usage),  Types of gynecological surgery | Case-control study | 477/479 | Hospital-based controls | cases 56.44 (±10.83) controls56.51 (±10.72) | Adenocarcinoma, Squamous cell carcinoma, Small cell carcinoma, Others | Overall lung cancer (Adenocarcinoma, Squamous cell carcinoma, Small cell carcinoma, Others) | Never smoker,  Ever smoker | only adjusted in model | Age, BMI, education, occupation, marital  status, tobacco smoking, passive smoking, alcohol drinking, exposure to cooking  oil fume (COF), tea consumption, history of lung disease, family history  of cancer surgery |
| Khedher, SB. et al. 2017 (6) | Mixed | Italy, Germany, USA, Canada | Age of menarche, Age at natural menopause, Menopause status, Menopause reason, Oophorectomy, Age at first child, Parity, Number of children, Breastfeeding | Case-control study | 4386/4177 | Hospital and population-based control | cases 63.3 years (range: 26–93)  controls 59.6 years (range: 20–97) | adenocarcinomas, squamous cell carcinomas, small cell lung cancers | Overall lung cancer (adenocarcinomas, squamous cell carcinomas, small cell lung cancers) | Never smoker,  Former smoker,  Current smoker | Never smoker,  Former smoker,  Current smoker | Study center, Age at interview or diagnosis, Ethnicity, Education, Smoking status, Comprehensive Smoking Index, Histological type |
| Patel, MI. et al. 2016(7) | Mixed | USA | Estrogen use, hormone therapy, OC use | Cohort study | 1044/129,951 | Participants in the Women’s Health Initiative Study | 50-79 | Overall lung cancer | Overall lung cancer | Never smoker,  Former smoker,  Current smoker | - | - |
| Schwartz, AG. et al. 2015 (8) | Mixed | USA | Parity, Number of live births, Age at first birth, Age at menopause, Years since menopause, Bilateral oophorectomy, Duration of previous, estrogen + progestin use | Cohort study | 2467 /160855 | Participants in the Women's Health Initiative (WHI) studies | 50-79 years | non-small cell lung cancers and small cell lung cancers | Overall lung cancer (non-small cell lung cancers and small cell lung cancers) | Never smoker,  Former smoker,  Current smoker | Never smoker,  Former smoker,  Current smoker | Age at screening, race/ethnicity, pack-years of smoking, education, US region, history of emphysema, history of asthma, BMI, family history of cancer, parity, number of live births, age at first live birth, age at menopause, years since menopause, bilateral oophorectomy, and duration of previous estrogen + progestin use |
| Tan, HS. et al. 2015(9) | Asian | Singapore | ages at menarche and menopause, pregnancy and delivery histories, ages at first and last delivery, breastfeeding history, the use of exogenous hormones | Cohort study | 311/28222 (446182 person years) | Participants in Singapore Breast Cancer Screening Project | 50-64 years | adenocarcinomas and non-adenocarcinomas | Overall lung cancer (adenocarcinomas and non-adenocarcinomas) | Never smoker,  Ever smoker | Never smoker,  Ever smoker | age at entry, ethnicity, body mass index, smoking status |
| Gallagher, L. et al. 2013 (10) | Asian | China | Pregnancy, Number of Pregnancies, Live Birth History, Age at First Live Birth, Duration of Breastfeeding, IUD, Tubal Ligation, Age at Menarche, Cause of Menopause, Age at Menopause, Hysterectomy, Oophorectomy, Reproductive Surgical Procedure | Cohort study | 824/267400 (2477861 person years) | Population-based control | ≥30 | - | Overall lung cancer | Never smoker,  Ever smoker | only adjusted in model | Age at baseline, smoking, parity, and quartile of endotoxin exposure |
| Pesatori, AC. et al. 2013 (11) | Caucasian | Italy | age at  menarche, cycle duration, parity,  age at first live-birth breastfeeding, menopausal status, age at menopause, ovariectomy, OC and HRT, duration of the reproductive period, duration of menopause, age at  diagnosis/enrollment. | Case-control study | 407 /499 | Population-based control | cases 65 (±10) controls 64 (±10) | adenocarcinomas and non-adenocarcinomas | Overall lung cancer (adenocarcinomas and non-adenocarcinomas) | Never smoker,  Ever smoker | only adjusted in model | area, age at study, smoking, environmental tobacco smoke (ETS), education, BMI |
| Lo, YL. et al. 2013 (12) | Asian | China | contraceptive medicine, hormone replacement therapy  (HRT) | Case-control study | 1540/1540 | 1:1 hospital-based matched case–control  study | Cases 58.38 (11.66)  Controls 58.94 (11.70) | Adenocarcinoma, squamous cell carcinoma, large cell carcinoma, small cell carcinoma, other unspecified carcinoma | Overall lung cancer | **Only never smokers** | **Only never smokers** | age and years of education |
| Lim, W. et al. 2012 (13) | Asian | Singapore | Parity (no. of children),  Age at first birth, Reproductive period,  Age at menarche,  Age at menopause,  Menstrual cycle length, Exogenous reproductive  hormone use | Case-control study | 702/1578 | hospital-based controls | Cases 65.9 (±11.8)  Controls 64.1 (±12.3) | Adenocarcinoma, squamous cell carcinoma, small cell carcinoma, bronchioalveolar, and not otherwise specified (NOS) | Overall lung cancer | Never smoker,  Ever smoker | Never smoker,  Ever smoker | Age at diagnosis, country of birth, housing type, number of years of education, smoking status, second-hand smoke exposure, history of cancer in a first-degree relative, mean intake of fruits and vegetables, a variable to indicate which case-control study the participant belonged to, smoking duration. |
| Brinton, LA. et al. 2012 (14) | Mixed | USA | ET-only use, EPT Use | Cohort study | 2097/118,008  (1,100,627 person-years.) | Participants in NIH-AARP Diet and Health Study Cohort (the second questionnaire) | 50–71 years | Adenocarcinoma, squamous cell, non-small cell, not otherwise specified (NOS), undifferentiated large cell | Overall lung cancer (Small Cell, Adenocarcinoma, Squamous Cell, Non-small cell, Undifferentiated/ Large cell) | Never smoker, Former smoker, Current smoker | Never smoker, Former smoker, Current smoker | age, race, age at menarche, menopausal type and age, BMI, emphysema diagnosis, cigarette smoking status and number of cigarettes/day, and other hormone therapy formulations. |
| Lin, Y. et al. 2012 (15) | Asian | China | Age at first birth  Age at menarche | Case-control study | 226/269 | Population and hospital based case-control study | - | Adenocarcinoma,  Squamous cell carcinoma,  Other | Overall lung cancer | Non-smokers, smokers | - | education, age, cooking oil fume, use of fume extractor, and ETS. |
| Meinhold, CL. et al. 2011 (16) | Mixed | USA | Age at menarche, Menopausal status, Number of live births, Age at first birth, Age at last birth, Oral contraceptives, Menopausal hormone therapy, Estrogen pills | Case-control study | 430/611 | Population controls and Hospital controls | Cases 66 Controls 64 | Non-small cell lung cancer | Non-small cell lung cancer | Never smoker, Former smoker, Current smoker | Never smoker, Former smoker, Current smoker | Age, education, smoking, number of smoking adults in household, current household income |
| Brinton, LA. et al. 2011 (17) | Caucasian | USA | Age at menarche, Parity, Number of births, Age at first live birth among parous women, Oral contraceptive use, Years of use of oral contraceptives, Natural menopause, Surgical menopause, bilateral oophorectomy, Surgical menopause, both ovaries intact, Menopausal hormone use, Years of use of menopausal hormones | Cohort study | 3512/185017  (1,816,356 person-years) | Participants in The NIH-AARP Diet and Health Study Cohort (the baseline questionnaire) | Cases 62.6 and controls 64.4 years (50-71) | SCLC, Adenocarcinoma, SCC, otherwise specified NOS, undifferentiated/large cell | Overall lung cancer (SCLC, Adenocarcinoma, SCC, otherwise specified NOS, undifferentiated/large cell) | Never smoker, Former smoker, Current smoker | Never smoker, Former smoker, Current smoker | age at entry into the cohort, race/ethnicity, education, body mass index, emphysema, smoking status and dose, age at menarche, and type of and age at menopause |
| Clague, J. et al. 2011 (18) | Mixed | USA | hormone therapy (HT) | Cohort study | 727/60592 | Participants in The California Teachers Study | Never HT user 65.6 ± 11.4  Former HT user 67.5 ± 10.5  Recent HT user 59.7 ± 9.4 | - | Overall lung cancer | Never smoker,  Ever smoker | Never smoker,  Ever smoker | race, smoking status/pack-years combination (never, former light, former heavy, current light, current heavy), type of menopause and BMI |
| Paulus, J. et al. 2010 (19) | Caucasian | USA | Parity, age at First Birth, number of live births | Case-control study | 1,004/848 | Participants in the Lung Cancer Susceptibility Study (LCSS) | cases 66.2 (10.8)  controls 58.4 (11.4) | Adenocarcinoma, squamous-cell carcinoma, bronchioalveolar carcinoma | Overall lung cancer | Never smoker,  Ever smoker | Never smoker,  Ever smoker | Age, smoking status, pack-years of smoking, and years since quitting smoking. |
| Baik, CS. et al. 2010 (20) | Caucasian | USA | age at menarche, age at menopause, type of menopause, parity, age at first birth, oral contraceptive use and postmenopausal  hormone (PMH) use | Cohort study | 1729/107,171 (1,590,432 person-years) | Participants in the Nurses’ Health Study | 63 (38-87) | adenocarcinoma, small  cell carcinoma, squamous cell carcinoma, large cell, unspecified non-small cell  lung cancer and other histologies | Overall lung cancer (adenocarcinoma, small  cell carcinoma, squamous cell carcinoma) | Never smoker, Former smoker, Current smoker | Never smoker, Former smoker, Current smoker | age at menopause, age at menarche, parity, type of menopause, PMH use, OCP use, smoking status, age at start smoking, cigarettes per day, time since quitting, fruit/vegetable intake, body mass index, environmental smoking exposure (parents smoking, years living with someone who smokes, exposure to smoking at work, exposure to smoking at home) |
| Chlebowski, RT. et al. 2010 (21) | Mixed | USA | Estrogen alone | RCT  (a randomised, double-blind, placebo-controlled trial) | Estrogen alone  group, 5310, case=61  Placebo group,  5429, case=54 | Placebo | 50-79 | Non-small-cell lung cancer, Small-cell lung cancer | Overall lung cancer (Non-small-cell lung cancer, Small-cell lung cancer) | Never smokers, past smokers, current smokers | - | stratified according to age, previous lung cancer, and dietary modification trial randomization |
| Slatore, CG. et al. 2010 (22) | Caucasian | USA | hormone replacement therapy (HRT) use | Cohort study | 344/36,588 | Participants in Vitamins and Lifestyle [VITAL] Study | 50 to 76 | non–small-cell lung cancer; small-cell lung cancer | Overall lung cancer  (non–small-cell lung cancer; small-cell lung cancer) | Never / current smokers | years smoked (only adjusted in model) | age, pack-years, pack-years squared, years smoked, personal history of cancer, family history of lung cancer, chronic obstructive pulmonary disease,  body mass index, age at menopause, hysterectomy type, and non-white race/ethnicity. |
| Brenner, DR. et al, 2010 (23) | Caucasian | Canada | Oral contraceptive use, Hormone replacement therapy, HRT type | Case-control study | 235/558 | Population and hospital-based control | 20-84 | Adenocarcinoma, Squamous cell carcinoma, Small-cell carcinoma, Large cell carcinoma,  Others/Mixed, Not classified/clinical  diagnosis | Overall lung cancer | Never smoker,  Ever smoker | - | Ethnicity |
| Heck, JE. et al, 2009 (24) | Caucasian | USA | Oral contraceptive use, Hormone replacement therapy | Case-control study | 150/148 | Population based control | Cases 61.9 ± 9.1  Controls 61.0 ± 10.2 | Squamous cell carcinoma, Small-cell carcinoma | Overall lung cancer | Never smoker,  Ever smoker | - | Age |
| Seow, A. et al. 2009 (25) | Asian | Singapore | Number of live births  Age at menopause  Age at menarche  Use of hormonal contraceptives, HRT | Cohort study | 298 /35,298 | Participants in the Singapore Chinese Health Study | 45-74 | adenocarcinomas and non-adenocarcinomas | Overall lung cancer (adenocarcinomas and non-adenocarcinomas) | Never smoker,  Ever smoker | Never smoker,  Ever smoker | age at interview, year of interview, dialect group, educational level, body mass index, total vegetable intake, total fruit/juice intake, β-cryptoxanthin, total isothiocyanates, and (except for nonsmokers) duration of smoking, cigarettes per day, and number of years since quitting. |
| Dorjgochoo, T. et al. 2009 (26) | Asian | China | oral contraceptives (OC), intrauterine devices (IUD) and tubal sterilization (TS) | Cohort study | 229/66661 | Participants in The Shanghai Women’s Health Study (population-based prospective study) | 40–70 years | - | Overall lung cancer | Never smoker,  Ever smoker | only adjusted in model | Education, age at menarche, number of live births, cumulative breastfeeding months, BMI, exercised regularly in past 5 years, smoking, menopausal status, first-degree family history of cancer, and other contraceptive methods |
| Chlebowski, RT. et al. 2009 (27) | Mixed | USA | Combined hormone  therapy | RCT  (a randomized, double-blind, placebo-controlled trial) | Combined hormone  therapy group n=8506 case=109, Placebo group n=8102 case=85  16 608 postmenopausal women | Placebo | 50-79 | Non-small-cell lung cancer, Small-cell lung cancer | Overall lung cancer (Non-small-cell lung cancer, Small-cell lung cancer) |  | - | Stratified according to  age, previous lung cancer, and randomization assignment in the dietary modification trial. |
| Smith, JR. et al 2009 (28) | Caucasian | USA | Hormone use | Cohort study | 87/2861 | Participants in the Rancho Bernardo  cohort study | 31 to 79 years | - | Overall lung cancer | Never smoker, Former smoker, Current smoker | only adjusted in model | Age, BMI, Education category, Marital status, Smoking status |
| Koushik, A. et al. 2009 (29) | Caucasian | Canada | age at first and last menstrual period, the reason for cessation of menstrual  periods, ovary removal status, age at oophorectomies, age at first pregnancy and first live birth, number of pregnancies and live births, and lactation history | Case-control study | 422/577 | Population-based  case-control study | Cases 61.5 (9.3), Controls 61.5 (9.4) | Adenocarcinoma, squamous cell carcinoma, small cell carcinoma, and large cell carcinoma | Overall lung cancer  (Adenocarcinoma) | Never smoker, Former smoker, Current smoker | comprehensive smoking  index (CSI) (only adjusted in model) | age, respondent status, ethnic group, number of years of schooling, mean census tract family income, and smoking  (represented by the CSI) |
| Rosenblatt, KA. et al. 2009 (30) | Asian | China | Oral contraceptives, Length of use of oral  contraceptives | Cohort study | 828/258956  2,410,072 person-years | Participants in this cohort study | 31-66 at cohort entry | - | Overall lung cancer | - | - | parity and age |
| Weiss, J. et al. 2008 (31) | Asian | China | Menopausal status, age at menarche, age at menopause, reproductive period, parity, age at first birth, intrauterine device use, oral contraceptive use, and postmenopausal hormone use. | Cohort study | 220 / 71,314 (506,522 person-years) | Participants in the prospective Shanghai  Women’s Health Study. | 40–70 | adenocarcinomas and non-adenocarcinomas | Overall lung cancer  (Adenocarcinoma) | Only lifetime nonsmokers | Only lifetime nonsmokers (stratified by passive smokers and non-passive smokers) | Passive smoke exposure (yes/no) |
| Ramnath, N. et al. 2008 (32) | Caucasian | USA | Hormone replacement therapy (HRT) | Case-control study | 595/1195 | hospital-based case-control study | Cases  61.3 [30–87]  Controls 61.0 [30–94] | Overall lung cancer | Overall lung cancer | Never smoker, Former smoker, Current smoker | Never smoker, Former smoker, Current smoker | age, smoking status, BMI, education and race |
| Rodriguez, C. et al. 2008 (33) | Caucasian | USA | Postmenopausal hormone therapy use | Cohort study | 659/72,772 | Participants in the Cancer Prevention Study II Nutrition Cohort. | 50-74 | Adenocarcinoma, squamous, large cell carcinomas, small cell carcinomas | Overall lung cancer | Never smoker, Former smoker, Current smoker | Never smoker, Former smoker, Current smoker | age at interview, smoking status, spousal tobacco exposure in 1992, body mass index in 1992, age at menopause, education, weekly servings of fruit, physical activity, total h-carotene intake, and oral contraceptive use. |
| Corrao, G. et al. 2008 (34) | Caucasian | Italy | Hormone replacement therapy (HRT) | Cohort study | 124/73505 | a large population-based prospective cohort  study | 45-75 | - | Overall lung cancer | - | - | - |
| Mahabir, S. et al. 2008 (35) | Mixed | USA | Hormone replacement therapy (HRT) | Case-control study | 763/838 | A hospital-based case-control study | Cases 60.75 Controls 60.11 | - | Overall lung cancer | Never smoker, Former smoker, Current smoker | - | - |
| Schwartz, AG. et al. 2007 (36) | Mixed | USA | Age at menarche, Age at menopause, Years of menses Cycles usually or always regular, Age at first live birth, No. of pregnancies, No. of children, Ever used OCs, Quartile of OC duration of use, Ever used HRT, Quartile of HRT duration of use, Quartile of estrogen, Ever used estrogen HRT alone, Ever used combined estrogen and progesterone HRT | Case-control study | 488/498 | Population-based control | 18-74 | Non–small-cell lung cancer | Non–small-cell lung cancer | Never smoker, Former smoker, Current smoker | Never smoker, Former smoker, Current smoker | Adjusted for age at diagnosis/interview, race, pack-years, family history of lung cancer, current body mass index, personal history of chronic obstructive lung disease, years exposed to passive smoke in the workplace, and education level. |
| Kabat, GC. et al. 2007 (37) | Caucasian | Canada | Parity, Number of live births, Age at first live birth, Age at menarche, Oral Contraceptive Use, Duration of OC use, Hormone Replacement Use, Duration of HRT use | Cohort study | 750/89812 | the Canadian National Breast  Screening Study (NBSS) | 40-59 years (at baseline)  Cases 50.8 (5.4) Non-cases 48.5 (5.6) | Squamous cell/epidermoid, adenocarcinoma, small cell, large cell, others | Overall lung cancer  (adenocarcinomas and non-adenocarcinomas) | Never smoker,  Ever smoker | Never smoker,  Ever smoker | Parity, age at menarche, age at first birth, menopausal status, oral contraceptive use, hormone replacement use, body mass index, education, smoking status, pack-years of smoking, study center and randomization group (intervention versus control) |
| Matsuo, K. et al. 2007 (38) | Asian | Japan | Fertile life, Age at menarche, Menopause Status, Age at menopause, Pregnancy, Age at first parity | Case-control study | 435/2175 (women 175/875) | Hospital-based design | *EGFR^mut^* 62.7±9.7 *EGFR^wt^*  62.5 ±9.4 Controls  62.6±9.4 | non-small-cell lung cancers | non-small-cell lung cancers  (EGFR-mutated  NSCLC, EGFR-wild-type NSCLC) | Never smoker, Former smoker, Current smoker | - | age |
| Chen, KY. et al. 2007 (39) | Asian | China | Hormone replacement therapy (HRT) | Case-control study | 826/531 | hospital-based case-control study | Cases 59.73 (12.30)  Controls 57.40 (14.16) | Non-small cell carcinoma, Adenocarcinoma, Squamous cell, Large cell, Small cell carcinoma | Overall lung cancer (Non-small cell carcinoma, Adenocarcinoma, Squamous cell, Large cell, Small cell carcinoma) | Never Smokers,  Ever Smokers | Never Smokers,  Ever Smokers | age, ethnicity, smoking, education, BMI, menopause, cooking, motorcycle riding, passive  smoking, incense burning, family history. The variable analyzed was not put into the multivariate  logistic regression model. |
| Elliott, A. et al. 2006 (40) | Caucasian | UK | Parity, Oral contraception status, Duration of oral contraception use, Time since last use of oral contraception, Time since first use of oral contraception, HRT status | Case-control study | 162/486 | nested case-control  Royal College of General Practitioners’ Oral  Contraception Study (OCS). | 29 (at recruitment) | - | Overall lung cancer |  | Daily cigarette consumption: 0, 1–14 or ≥15)  (only adjusted in model) | smoking, social class and parity except where the variable itself is being examined. |
| Gorlova, OY. et al. 2006 (41) | Mixed | USA | Parity, OC and hormone use, menopausal status, age at menopause, miscarriages history | Case-control study | 189/177 | Hospital-based control | Cases 60.2 ± 12.7  Controls 61.9 ± 10.9 | Adenocarcinoma, bronchioalveolar carcinoma, small cell carcinoma, squamous cell carcinoma, non-small cell lung cancer | Overall lung cancer | **Only never smokers** | **Only never smokers** | Age, gender, ethnicity, years of education, and income |
| Liu, Y. et al. 2005 (42) | Asian | Japan | Menopausal status Hormone use, Breast feeding, Age at menarche, Age at menopause, Years of menstruation, Parity, Age at first live birth | Cohort study | 153/44,677 | population-based  The Japan Public Health Center-based Prospective Study (JPHC  Study) | 40-69 (at baseline) | Overall lung cancer | Overall lung cancer | **Only lifelong never-smoking women** | **Only lifelong never-smoking women** | Age, public health center (PHC) area, and passive smoking during childhood or in the workplace. |
| Schabath, B. et al. 2004 (43) | Mixed | USA | hormone replacement therapy | Case-control study | 499/519 | Healthy women without a previous diagnosis of cancer was recruited from the Kelsey-Seybold  Clinics, Houston’s largest private multispecialty physician  Group. Matching criteria were age, gender, ethnicity, and smoking  status (never, former, or current). | Case 59.7 (10.3)  Controls 58.6 (10.7) | non–small-cell lung cancer, small-cell lung  cancer | Overall lung cancer  (non–small-cell lung cancer, small-cell lung  cancer) | Never smoker, Former smoker, Current smoker | Never smoker, Former smoker, Current smoker | ethnicity, smoking status, education, BMI, and menopausal status. |
| Kreuzer, M. et al. 2003 (44) | Caucasian | Germany | Age at first menarche, Length of the menstrual cycle, Age at first pregnancy, No. of full-term pregnancies, Menopausal status, Age at natural menopause, Use of oral contraceptives, Use of hormones, HRT | Case-control study | 811/912 | Population based case-control study | Case 60 Control 59 | Adenocarcinoma,  Small cell lung cancer, Squamous cell carcinoma | Overall lung cancer (Adenocarcinoma,  Small cell lung cancer, Squamous cell carcinoma) | Never smoker,  Ever smoker | Never smoker,  Ever smoker | age, region, log(packyear+1), smoking, and educational level |
| Olsson, H. et al. 2003 (45) | Caucasian | Sweden | Hormone replacement therapy (HRT) | Cohort study | 55/29,508  (226,611 person-years) | women aged 25–65 years were randomly selected from the general population of the South Swedish Health Care Region. Eligible women were born in Sweden and had no history of cancer. | 25–65 years | - | Overall lung cancer | Never smoker,  Ever smoker | - | Calendar year and 5-year age group |
| Zatloukal, P. et al. 2003 (46) | Caucasian | Czech Republic | No. of deliveries, No. of miscarriages, Age at menarche, Cycle (repeat) of menses, Duration of menstrual flow, Quantity of menstrual flow, Onset of menopause, Mental tension or pains related to menses | Case-control study | 145 cases of adenocarcinoma of the lung, 221 lung cancer cases of other cell types, 1624 controls | A hospital-based case-control study | 25-89  case 62.8 (10.1) controls 57.0 (13.0) | Adenocarcinoma, Squamous/small/large-cell | Adenocarcinoma, Squamous/small/large-cell | Never smoker, past-smokers, current smokers | only adjusted in model | age, residence, education and pack-years of smoking |
| Brenner, A. et al. 2003 (47) | Asian | China | Age at menarche, Age at menopause, Menstrual cycle length, Length of menstrual flow, number of pregnancies, Age at first live births, Number of live births, Number of menstrual cycles up to the reference date | Case-control study | 109/435 | a population based case-control study | 30-75 | Small cell carcinomas, squamous cell carcinomas, adenocarcinoma, and pulmonary neoplasms | Overall lung cancer | Never smoker,  Ever smoker | - | age and prefecture. |
| Hulley, S. et al. 2002 (48) | Caucasian | USA | Conjugated Estrogen + Progestin | RCT  (a randomized, blinded, placebo-controlled trial) | Case: 37/27  Hormone group 1380 / placebo group 1383  Total: 5084 | placebo | 67(7) | - | Overall lung cancer | - | only adjusted in model | Age, education, current, and former smoking, BMI, low-density, lipoprotein, cholesterol, and digitalis use. |
| Seow, A. et al. 2002 (49) | Asian | Singapore | Number of livebirths,  Age at menarche, Age at first birth,  Age at menopause, Menstrual cycle length | Case-control study | 303/765 | Hospital-based control | 20-89 | Adenocarcinomas, squamous cell, small cell carcinomas, large cell undifferentiated | Overall lung cancer | Lifetime non-smokers,  smokers | Lifetime non-smokers,  smokers | Age, number of live births, family history of cancer. For smokers, adjusted additionally for duration and intensity. For non-smokers, further adjustment for passive smoking did not materially affect estimates. |
| Kubik, AK. et al. 2002 (50) | Caucasian | Czech Republic | No. of deliveries, No. of miscarriages, Age at menarche, Cycle (repetition) of menses, Duration of menstrual flow, Quantity of menstrual flow, Onset of menopause (years), Mental tension or pains related to menses | Case-control study | 269/1079 | A hospital-based case–control study | 25-89  Cases 63.7 (10.7)  Controls 57.2 (13.8) | Squamous cell, small cell, adenocarcinoma, bronchioloalveolar, large cell, carcinoma not otherwise specified. | Overall lung cancer (Squamous + small + large cell, Adenoca + bronchioloalveolar carcinoma) | Never smoker, Former smoker, Current smoker | - | adjusted for age, residence, education, and pack-years of smoking. |
| Blackman, JA. et al. 2002 (51) | Mixed | USA | Estrogen replacement therapy | Case-control study | 662/4671 | Hospital-based Case-Control Surveillance Study | Case 40-74 | Adenocarcinoma, small cell/oat cell, squamous, other cell types | Overall lung cancer  (Adenocarcinoma, Small cell/oat cell, Squamous, Other) | Never smoker, Former smoker, Current smoker | only adjusted in model | age, year of interview, study center, education, race, duration of cigarette smoking, and number of cigarettes smoked per day. |
| Rossouw, JE. et al. 2002 (52) | Mixed | USA | Estrogen plus progestin use | RCT | Case:54/50  Estrogen + Progestin group=8506  Placebo=8102  Total=16608 | Placebo | 50-79 | - | Overall lung cancer | Never smokers, past smokers, current smokers | - | Clinical center, age, prior disease, and randomization status in the low-fat diet trial |
| Pukkala, E. et al. 2001 (53) | Caucasian | Finland | Long cycle and monthly cycle hormonal replacement therapy (HRT) | Cohort study | 55/94505 | Participants in this cohort study | - | - | Overall lung cancer | - | - |  |
| Zhou, BS. et al. 2000 (54) | Asian | China | Age of first  Menstruation, Menstruation  cycle (days), Number of live births | Case-control study | 72/72 | 1:1 matched population based case-control study | Case 56.6  Control 57.2 | Adenocarcinoma | Adenocarcinoma | - | - | Cooking fumes, family history of lung cancer, economic income, no. of live births, intake of vitamin E and β-carotene |
| Persson, I. et al. 1996 (55) | Caucasian | Sweden | Hormone replacement therapy (HRT) | Cohort study | 112/22597 | Participants in this cohort study | mean age of 54.5 years at cohort entry | - | Overall lung cancer | - | - | - |
| Taioli, E. et al. 1994 (56) | Mixed | USA | Age at menarche, age at first  pregnancy, number of full-term  pregnancies, oral contraceptives,  estrogen replacement, breast  feeding, cycle length, period length, age at menopause,  type of menopause | Case-control study | 180/303 | hospital-based case-control study | 20-89 | Adenocarcinoma | Adenocarcinoma | Never smoker, Former smoker, Current smoker | only adjusted in model | smoking,  age at diagnosis, years of education, body mass index, plus menopausal status, type of menopause |
| Wu-Williams, AH. et al. 1990 (57) | Asian | China | Age at menarche, Number of children, Age at natural menopause, Hysterectomy, Miscarriage,  Spont. abortion,  Difficult labor,  Oral contraceptive | Case-control study | 965 / 959 | a population based case-control study | - | Adenocarcinoma, squamous cell carcinomas, oat/small cell carcinomas, large cell carcinomas, other known cell types | Overall lung cancer | Smokers and non-smokers | only adjusted in model | age, education, personal smoking and study area. |
| Adami, HO. et al. 1989 (58) | Caucasian | Sweden | non-contraceptive estrogens | Cohort study | 46/23,244 | Participants in the a population-based cohort | 54.5 at cohort entry | - | Overall lung cancer | Never smoker, Former smoker, Current smoker | - | Person-years and the age-specific incidence rates per calendar year and 5-year age group |
| Wu, AH. et al. 1988 (59) | Caucasian | USA | Age at menarche, menopause status, age at natural menopause, oral contraceptive, hormones | Case-control study | 336/336 | 1:1 population based case-control study | Cases 59.4 Controls 59.2 | Adenocarcinoma | Adenocarcinoma | Current smokers, former smokers | only adjusted in model | Pack-years of smoking, years since smoking stopped, and depth of inhalation |
| Gao, YT. et al. 1987 (60) | Asian | China | Length of menstrual  cycle | Case-control study | 672/735 | a population based case-control study | 35-69 | Squamous, Adenocarcinoma | Overall lung cancer (Squamous, Adenocarcinoma) | Smokers and non-smokers | only adjusted in model | age. education, smoking and regularity of menstruation |
| Chinese: | | | | | | | | | | | | |
| Xiang, YB. et al. 2003(61) | Asian | China | Number of live births | Case-control study | 504/601 | a population based case-control study | 35-69 | - | Overall lung cancer | **Non-smokers** | **Non-smokers** | Age, BMI, income |
| Chen, W. et al. 2009 (62) | Asian | China | Age at menarche, Cycle usually or always regular, Menopause Status, Menopause type, Age at menopause, number of pregnancies, OC, Age at first use of OC, OC duration, HRT, Intrauterine device use | Cohort study | 271/72829 | The Shanghai Women’s Health Study | 51.5±8.37 | - | Overall lung cancer | **Non-smokers** | **Non-smokers** | Age, education, income |
| Lin, Y. et al. 2010(63) | Asian | China | age at menarche | Case-control study | 208/208 | 1:1 population based case-control study | >20 | Squamous cell, small cell, adenocarcinoma, bronchioloalveolar, large cell, carcinoma not otherwise specified | Overall lung cancer | - | - | Indoor cooking, fruits, passive smoking, tea, application of pesticide, activity |
| Zheng, W. et al. 1988(64) | Asian | China | Menstrual cycle length, Age at menarche, Age at menopause, Reproductive Surgical Procedure | Case-control study | 672/735 | a population based case-control study | 35-69 | Squamous cell, small cell, adenocarcinoma, others | Overall lung cancer (Adenocarcinoma, Squamous/small cell) | Smokers and non-smokers | only adjusted in model | Age, smoking and regular menstrual cycle |
| Yin, Z. et al. 2005(65) | Asian | China | Age at first pregnancy, number of pregnancies, Age at menopause, OC | Case-control study | 409/436 | a population based case-control study | 35-69 | Squamous cell, small cell, adenocarcinoma, others | Overall lung cancer | **Non-smokers** | **Non-smokers** | Age at first pregnancy, number of pregnancies, Age at menopause, OC |
| Li, X. et al. 1995 (66) | Asian | China | OC | Case-control study | 79/79 | 1:1 population based case-control study | 28-76 | Squamous cell, small cell, adenocarcinoma, others | Overall lung cancer | Smokers and non-smokers | - | - |

**Supplementary Table S3. Quality assessment of studies using the Newcastle-Ottawa scale**

| **Study** | **Study design** | **Average score from two independent reviewers** |
| --- | --- | --- |
| Vohra, SN. et al. 2019 [1] | Case-control study | 6.5 |
| Jin, K. et al. 2019[2] | Case-control study | 6.5 |
| Titan, AL. et al. 2019[3] | Cohort study | 7 |
| Iversen, L. et al. 2017 [4] | Cohort study | 7 |
| He, F. et al. 2017 [5] | Case-control study | 5 |
| Khedher, SB. et al. 2017 [6] | Case-control study | 5.5 |
| Patel, MI. et al. 2016[7] | Cohort study | 7 |
| Schwartz, AG. et al. 2015 [8] | Cohort study | 7 |
| Tan, HS. et al. 2015[9] | Cohort study | 8 |
| Gallagher, L. et al. 2013 [10] | Cohort study | 7 |
| Pesatori, AC. et al. 2013 [11] | Case-control study | 7.5 |
| Lo, YL. et al. 2013 [12] | Case-control study | 7 |
| Lim, W. et al. 2012 [13] | Case-control study | 7 |
| Brinton, LA. et al. 2012 [14] | Cohort study | 7 |
| Lin, Y. et al. 2012 [15] | Case-control study | 5.5 |
| Meinhold, CL. et al. 2011 [16] | Case-control study | 6 |
| Brinton, LA. et al. 2011 [17] | Cohort study | 7 |
| Clague, J. et al. 2011 [18] | Cohort study | 7 |
| Paulus, J. et al. 2010 [19] | Case-control study | 7 |
| Baik, CS. et al. 2010 [20] | Cohort study | 8 |
| Chlebowski, RT. et al. 2010 [21] | RCT | - |
| Slatore, CG. et al. 2010 [22] | Cohort study | 6 |
| Brenner, DR. et al, 2010 [23] | Case-control study | 7.5 |
| Heck, JE. et al, 2009 [24] | Case-control study | 7.5 |
| Seow, A. et al. 2009 [25] | Cohort study | 9 |
| Dorjgochoo, T. et al. 2009 [26] | Cohort study | 8 |
| Chlebowski, RT. et al. 2009 [27] | RCT | - |
| Smith, JR. et al 2009 [28] | Cohort study | 7 |
| Koushik, A. et al. 2009 [29] | Case-control study | 6 |
| Rosenblatt, KA. et al. 2009 [30] | Cohort study | 7 |
| Weiss, J. et al. 2008 [31] | Cohort study | 7 |
| Ramnath, N. et al. 2008 [32] | Case-control study | 6 |
| Rodriguez, C. et al. 2008 [33] | Cohort study | 8 |
| Corrao, G. et al. 2008 [34] | Cohort study | 4.5 |
| Mahabir, S. et al. 2008 [35] | Case-control study | 7 |
| Schwartz, AG. et al. 2007 [36] | Case-control study | 6 |
| Kabat, GC. et al. 2007 [37] | Cohort study | 7 |
| Matsuo, K. et al. 2007 [38] | Case-control study | 6 |
| Chen, KY. et al. 2007 [39] | Case-control study | 6 |
| Elliott, A. et al. 2006 [40] | Case-control study | 6 |
| Gorlova, OY. et al. 2006 [41] | Case-control study | 7 |
| Liu, Y. et al. 2005 [42] | Cohort study | 8 |
| Schabath, B. et al. 2004 [43] | Case-control study | 7 |
| Kreuzer, M. et al. 2003 [44] | Case-control study | 6 |
| Olsson, H. et al. 2003 [45] | Cohort study | 6 |
| Zatloukal, P. et al. 2003 [46] | Case-control study | 5.5 |
| Brenner, A. et al. 2003 [47] | Case-control study | 6.5 |
| Hulley, S. et al. 2002 [48] | RCT | - |
| Seow, A. et al. 2002 [49] | Case-control study | 6 |
| Kubik, AK. et al. 2002 [50] | Case-control study | 6 |
| Blackman, JA. et al. 2002 [51] | Case-control study | 5 |
| Rossouw, JE. et al. 2002 [52] | RCT | - |
| Pukkala, E. et al. 2001 [53] | Cohort study | 5 |
| Zhou, BS. et al. 2000 [54] | Case-control study | 7 |
| Persson, I. et al. 1996 [55] | Cohort study | 6 |
| Taioli, E. et al. 1994 [56] | Case-control study | 5 |
| Wu-Williams, AH. et al. 1990 [57] | Case-control study | 6 |
| Adami, HO. et al. 1989 [58] | Cohort study | 5 |
| Wu, AH. et al. 1988 [59] | Case-control study | 6 |
| Gao, YT. et al. 1987 [60] | Case-control study | 6 |
| Xiang, YB. et al. 2003[61] | Case-control study | 5.5 |
| Chen, W. et al. 2009 [62] | Cohort study | 7 |
| Lin, Y. et al. 2010[63] | Case-control study | 5.5 |
| Zheng, W. et al. 1988[64] | Case-control study | 6 |
| Yin, Z. et al. 2005[65] | Case-control study | 5 |
| Li, X. et al. 1995 [66] | Case-control study | 5.5 |

**Supplementary Table S4. The result of the first independent reviewer for quality assessment of studies using the Newcastle-Ottawa scale**

| **Study** | **Study design** | **Selection^a^** | **Comparability^b^** | **Outcome/Exposure^c^** | **Score** |
| --- | --- | --- | --- | --- | --- |
| Vohra, SN. et al. 2019 (1) | Case-control study | *** | ** | ** | 7 |
| Jin, K. et al. 2019(2) | Case-control study | **** | ** | * | 7 |
| Titan, AL. et al. 2019(3) | Cohort study | *** | ** | ** | 7 |
| Iversen, L. et al. 2017 (4) | Cohort study | *** | ** | ** | 7 |
| He, F. et al. 2017 (5) | Case-control study | *** | * | * | 5 |
| Khedher, SB. et al. 2017 (6) | Case-control study | ** | ** | * | 5 |
| Patel, MI. et al. 2016(7) | Cohort study | *** | ** | ** | 7 |
| Schwartz, AG. et al. 2015 (8) | Cohort study | *** | ** | ** | 7 |
| Tan, HS. et al. 2015(9) | Cohort study | **** | ** | ** | 8 |
| Gallagher, L. et al. 2013 (10) | Cohort study | *** | ** | ** | 7 |
| Pesatori, AC. et al. 2013 (11) | Case-control study | *** | ** | *** | 8 |
| Lo, YL. et al. 2013 (12) | Case-control study | *** | ** | ** | 7 |
| Lim, W. et al. 2012 (13) | Case-control study | *** | ** | ** | 7 |
| Brinton, LA. et al. 2012 (14) | Cohort study | *** | ** | ** | 7 |
| Lin, Y. et al. 2012 (15) | Case-control study | *** | ** | * | 6 |
| Meinhold, CL. et al. 2011 (16) | Case-control study | *** | ** | * | 6 |
| Brinton, LA. et al. 2011 (17) | Cohort study | *** | ** | ** | 7 |
| Clague, J. et al. 2011 (18) | Cohort study | *** | ** | ** | 7 |
| Paulus, J. et al. 2010 (19) | Case-control study | *** | ** | ** | 7 |
| Baik, CS. et al. 2010 (20) | Cohort study | *** | ** | *** | 8 |
| Chlebowski, RT. et al. 2010 (21) | RCT |  |  |  |  |
| Slatore, CG. et al. 2010 (22) | Cohort study | *** | ** | * | 6 |
| Brenner, DR. et al, 2010 (23) | Case-control study | *** | ** | ** | 7 |
| Heck, JE. et al, 2009 (24) | Case-control study | **** | ** | * | 7 |
| Seow, A. et al. 2009 (25) | Cohort study | **** | ** | *** | 9 |
| Dorjgochoo, T. et al. 2009 (26) | Cohort study | *** | ** | *** | 8 |
| Chlebowski, RT. et al. 2009 (27) | RCT |  |  |  |  |
| Smith, JR. et al 2009 (28) | Cohort study | *** | ** | ** | 7 |
| Koushik, A. et al. 2009 (29) | Case-control study | *** | ** | * | 6 |
| Rosenblatt, KA. et al. 2009 (30) | Cohort study | *** | ** | ** | 7 |
| Weiss, J. et al. 2008 (31) | Cohort study | **** | ** | * | 7 |
| Ramnath, N. et al. 2008 (32) | Case-control study | *** | ** | * | 6 |
| Rodriguez, C. et al. 2008 (33) | Cohort study | *** | ** | *** | 8 |
| Corrao, G. et al. 2008 (34) | Cohort study | *** |  | * | 4 |
| Mahabir, S. et al. 2008 (35) | Case-control study | *** | ** | ** | 7 |
| Schwartz, AG. et al. 2007 (36) | Case-control study | *** | ** | * | 6 |
| Kabat, GC. et al. 2007 (37) | Cohort study | *** | ** | ** | 7 |
| Matsuo, K. et al. 2007 (38) | Case-control study | *** | ** | * | 6 |
| Chen, KY. et al. 2007 (39) | Case-control study | *** | ** | * | 6 |
| Elliott, A. et al. 2006 (40) | Case-control study | *** | ** | * | 6 |
| Gorlova, OY. et al. 2006 (41) | Case-control study | *** | ** | ** | 7 |
| Liu, Y. et al. 2005 (42) | Cohort study | *** | ** | *** | 8 |
| Schabath, B. et al. 2004 (43) | Case-control study | *** | ** | ** | 7 |
| Kreuzer, M. et al. 2003 (44) | Case-control study | *** | ** | * | 6 |
| Olsson, H. et al. 2003 (45) | Cohort study | **** | * | * | 6 |
| Zatloukal, P. et al. 2003 (46) | Case-control study | ** | ** | * | 5 |
| Brenner, A. et al. 2003 (47) | Case-control study | *** | ** | * | 6 |
| Hulley, S. et al. 2002 (48) | RCT |  |  |  |  |
| Seow, A. et al. 2002 (49) | Case-control study | ** | ** | ** | 6 |
| Kubik, AK. et al. 2002 (50) | Case-control study | ** | ** | ** | 6 |
| Blackman, JA. et al. 2002 (51) | Case-control study | ** | ** | * | 5 |
| Rossouw, JE. et al. 2002 (52) | RCT |  |  |  |  |
| Pukkala, E. et al. 2001 (53) | Cohort study | *** | * | * | 5 |
| Zhou, BS. et al. 2000 (54) | Case-control study | **** | ** | * | 7 |
| Persson, I. et al. 1996 (55) | Cohort study | *** | * | ** | 6 |
| Taioli, E. et al. 1994 (56) | Case-control study | ** | ** | * | 5 |
| Wu-Williams, AH. et al. 1990 (57) | Case-control study | *** | ** | * | 6 |
| Adami, HO. et al. 1989 (58) | Cohort study | ** | ** | * | 5 |
| Wu, AH. et al. 1988 (59) | Case-control study | *** | ** | * | 6 |
| Gao, YT. et al. 1987 (60) | Case-control study | *** | ** | * | 6 |
| Chinese: | | | | | |
| Xiang, YB. et al. 2003(61) | Case-control study | *** | * | * | 5 |
| Chen, W. et al. 2009 (62) | Cohort study | *** | ** | ** | 7 |
| Lin, Y. et al. 2010(63) | Case-control study | *** | ** | * | 6 |
| Zheng, W. et al. 1988(64) | Case-control study | *** | ** | * | 6 |
| Yin, Z. et al. 2005(65) | Case-control study | ** | ** | * | 5 |
| Li, X. et al. 1995 (66) | Case-control study | ** | ** | * | 5 |

^a^Maximum of 4 stars; ^b^Maximum of 2 stars; ^c^Maximum of 4 stars;

**Supplementary Table S5. The result of the second independent reviewer for quality assessment of studies using the Newcastle-Ottawa scale**

| **Study** | **Study design** | **Selection^a^** | **Comparability^b^** | **Outcome/Exposure^c^** | **Score^d^** |
| --- | --- | --- | --- | --- | --- |
| Vohra, SN. et al. 2019 (1) | Case-control study | *** | ** | * | 6 |
| Jin, K. et al. 2019(2) | Case-control study | **** | * | * | 6 |
| Titan, AL. et al. 2019(3) | Cohort study | *** | ** | ** | 7 |
| Iversen, L. et al. 2017 (4) | Cohort study | *** | ** | ** | 7 |
| He, F. et al. 2017 (5) | Case-control study | *** | * | * | 5 |
| Khedher, SB. et al. 2017 (6) | Case-control study | *** | ** | * | 6 |
| Patel, MI. et al. 2016(7) | Cohort study | *** | ** | ** | 7 |
| Schwartz, AG. et al. 2015 (8) | Cohort study | *** | ** | ** | 7 |
| Tan, HS. et al. 2015(9) | Cohort study | **** | ** | ** | 8 |
| Gallagher, L. et al. 2013 (10) | Cohort study | *** | ** | ** | 7 |
| Pesatori, AC. et al. 2013 (11) | Case-control study | *** | ** | ** | 7 |
| Lo, YL. et al. 2013 (12) | Case-control study | *** | ** | ** | 7 |
| Lim, W. et al. 2012 (13) | Case-control study | *** | ** | ** | 7 |
| Brinton, LA. et al. 2012 (14) | Cohort study | *** | ** | ** | 7 |
| Lin, Y. et al. 2012 (15) | Case-control study | *** | * | * | 5 |
| Meinhold, CL. et al. 2011 (16) | Case-control study | *** | ** | * | 6 |
| Brinton, LA. et al. 2011 (17) | Cohort study | *** | ** | ** | 7 |
| Clague, J. et al. 2011 (18) | Cohort study | *** | ** | ** | 7 |
| Paulus, J. et al. 2010 (19) | Case-control study | *** | ** | ** | 7 |
| Baik, CS. et al. 2010 (20) | Cohort study | *** | ** | *** | 8 |
| Chlebowski, RT. et al. 2010 (21) | RCT |  |  |  |  |
| Slatore, CG. et al. 2010 (22) | Cohort study | *** | ** | * | 6 |
| Brenner, DR. et al, 2010 (23) | Case-control study | **** | ** | ** | 8 |
| Heck, JE. et al, 2009 (24) | Case-control study | **** | ** | ** | 8 |
| Seow, A. et al. 2009 (25) | Cohort study | **** | ** | *** | 9 |
| Dorjgochoo, T. et al. 2009 (26) | Cohort study | *** | ** | *** | 8 |
| Chlebowski, RT. et al. 2009 (27) | RCT |  |  |  |  |
| Smith, JR. et al 2009 (28) | Cohort study | *** | ** | ** | 7 |
| Koushik, A. et al. 2009 (29) | Case-control study | *** | ** | * | 6 |
| Rosenblatt, KA. et al. 2009 (30) | Cohort study | *** | ** | ** | 7 |
| Weiss, J. et al. 2008 (31) | Cohort study | **** | ** | * | 7 |
| Ramnath, N. et al. 2008 (32) | Case-control study | *** | ** | * | 6 |
| Rodriguez, C. et al. 2008 (33) | Cohort study | *** | ** | *** | 8 |
| Corrao, G. et al. 2008 (34) | Cohort study | *** |  | ** | 5 |
| Mahabir, S. et al. 2008 (35) | Case-control study | *** | ** | ** | 7 |
| Schwartz, AG. et al. 2007 (36) | Case-control study | *** | ** | * | 6 |
| Kabat, GC. et al. 2007 (37) | Cohort study | *** | ** | ** | 7 |
| Matsuo, K. et al. 2007 (38) | Case-control study | *** | ** | * | 6 |
| Chen, KY. et al. 2007 (39) | Case-control study | *** | ** | * | 6 |
| Elliott, A. et al. 2006 (40) | Case-control study | *** | ** | * | 6 |
| Gorlova, OY. et al. 2006 (41) | Case-control study | *** | ** | ** | 7 |
| Liu, Y. et al. 2005 (42) | Cohort study | *** | ** | *** | 8 |
| Schabath, B. et al. 2004 (43) | Case-control study | *** | ** | ** | 7 |
| Kreuzer, M. et al. 2003 (44) | Case-control study | *** | ** | * | 6 |
| Olsson, H. et al. 2003 (45) | Cohort study | **** | * | * | 6 |
| Zatloukal, P. et al. 2003 (46) | Case-control study | *** | ** | * | 6 |
| Brenner, A. et al. 2003 (47) | Case-control study | **** | ** | * | 7 |
| Hulley, S. et al. 2002 (48) | RCT |  |  |  |  |
| Seow, A. et al. 2002 (49) | Case-control study | ** | ** | ** | 6 |
| Kubik, AK. et al. 2002 (50) | Case-control study | ** | ** | ** | 6 |
| Blackman, JA. et al. 2002 (51) | Case-control study | ** | ** | * | 5 |
| Rossouw, JE. et al. 2002 (52) | RCT |  |  |  |  |
| Pukkala, E. et al. 2001 (53) | Cohort study | *** | * | * | 5 |
| Zhou, BS. et al. 2000 (54) | Case-control study | **** | ** | * | 7 |
| Persson, I. et al. 1996 (55) | Cohort study | *** | * | ** | 6 |
| Taioli, E. et al. 1994 (56) | Case-control study | *** | * | * | 5 |
| Wu-Williams, AH. et al. 1990 (57) | Case-control study | *** | ** | * | 6 |
| Adami, HO. et al. 1989 (58) | Cohort study | ** | ** | * | 5 |
| Wu, AH. et al. 1988 (59) | Case-control study | *** | ** | * | 6 |
| Gao, YT. et al. 1987 (60) | Case-control study | *** | ** | * | 6 |
| Chinese: | | | | | |
| Xiang, YB. et al. 2003(61) | Case-control study | **** | * | * | 6 |
| Chen, W. et al. 2009 (62) | Cohort study | *** | ** | ** | 7 |
| Lin, Y. et al. 2010(63) | Case-control study | *** | * | * | 5 |
| Zheng, W. et al. 1988(64) | Case-control study | **** | * | * | 6 |
| Yin, Z. et al. 2005(65) | Case-control study | ** | ** | * | 5 |
| Li, X. et al. 1995 (66) | Case-control study | *** | ** | * | 6 |

^a^Maximum of 4 stars; ^b^Maximum of 2 stars; ^c^Maximum of 4 stars;

**Supplementary Table S6: Cochrane Risk of Bias Tool for randomized controlled trials**

| **Study** | **Study design** | **First reviewer** | **Second reviewer** | **Average score** |
| --- | --- | --- | --- | --- |
| Chlebowski, RT. et al. 2010 [21] | RCT | 6 | 7 | 6.5 |
| Chlebowski, RT. et al. 2009 [27] | RCT | 5 | 6 | 5.5 |
| Hulley, S. et al. 2002 [48] | RCT | 5 | 5 | 5 |
| Rossouw, JE. et al. 2002 [52] | RCT | 5 | 5 | 5 |

**Supplementary Table S7. The result of the first independent reviewer of the Cochrane Risk of Bias Tool for randomized controlled trials**

| **Study** | **Study design** | Selection bias  (random sequence generation) | Selection bias  (Allocation concealment) | Reporting bias  (Selective reporting) | Other bias (Other sources of bias) | Performance bias (Blinding- participants and personnel) | Detection bias (Outcome assessment) | Attribution bias (Incomplete outcome data) | Total score* |
| --- | --- | --- | --- | --- | --- | --- | --- | --- | --- |
| Chlebowski, RT. et al. 2010 [21] | RCT | + | + | + | ? | + | + | + | 6 |
| Chlebowski, RT. et al. 2009 [27] | RCT | + | + | + | ? | + | + | ? | 5 |
| Hulley, S. et al. 2002 [48] | RCT | + | + | + | ? | + | ? | + | 5 |
| Rossouw, JE. et al. 2002 [52] | RCT | + | ? | + | ? | + | + | + | 5 |

*Total score: points awarded based on number of “+” or low risk of bias

+ = Low risk of bias, ? = Unclear risk of bias, - = High risk of bias

**Supplementary Table S8. The result of the second independent reviewer of the Cochrane Risk of Bias Tool for randomized controlled trials**

| **Study** | **Study design** | Selection bias  (random sequence generation) | Selection bias  (Allocation concealment) | Reporting bias  (Selective reporting) | Other bias (Other sources of bias) | Performance bias (Blinding- participants and personnel) | Detection bias (Outcome assessment) | Attribution bias (Incomplete outcome data) | Total score* |
| --- | --- | --- | --- | --- | --- | --- | --- | --- | --- |
| Chlebowski, RT. et al. 2010 [21] | RCT | + | + | + | + | + | + | + | 7 |
| Chlebowski, RT. et al. 2009 [27] | RCT | + | + | + | + | + | ? | + | 6 |
| Hulley, S. et al. 2002 [48] | RCT | + | + | + | + | ? | ? | + | 5 |
| Rossouw, JE. et al. 2002 [52] | RCT | + | ? | + | + | ? | + | + | 5 |

*Total score: points awarded based on number of “+” or low risk of bias

+ = Low risk of bias, ? = Unclear risk of bias, - = High risk of bias

**Supplementary Table S9. Sensitive analysis of the association of reproductive factors and overall lung cancer risk**

| Reproductive factors | Highest category | Lowest category (reference) | No. of studies | P_het_ ^†^ | *I^2^* value (%) | OR (95% CI)^‡^ |
| --- | --- | --- | --- | --- | --- | --- |
| Menstrual-related factors |  |  |  |  |  |  |
| Age at menopause | ≥50 to ≥55 | Premenopausal or <50 | 20 | <0.001 | 60.9 | 0.95 (0.84, 1.07) |
| Age at menarche | ≥15 to ≥18 | <11 to ≤15 |  | 0.032 | 40.4 | 1.03 (0.96, 1.10) |
| Non-natural menopause | **Non-natural** | **Natural / premenopausal** | **10** | **0.008** | **59.5** | **1.56 (1.26, 1.93)** |
| Ovariectomy | **yes** | **no** | **5** | **0.487** | **0.0** | **1.38 (1.16, 1.64)** |
| Hysterectomy | yes | no | 4 | 0.171 | 40.1 | 1.21 (0.98, 1.49) |
| Ovariectomy and Hysterectomy | yes | no | 3 | 0.256 | 26.7 | 1.24 (0.95, 1.61) |
| Menstrual cycle length | **>30 days** | **<27 to ≤ 30 days** | **7** | **0.074** | **50.1** | **0.77 (0.63, 0.95)** |
| Menopausal status | Post-menopausal | Pre-menopausal | 6 | 0.057 | 53.4 | 1.26 (0.92, 1.73) |
| Length of menstrual flow (days) | ≥5 to >6 | ≤3 to <5 | 3 | 0.759 | 0.0 | 0.98 (0.80, 1.20) |
| Other factors |  |  |  |  |  |  |
| Hormone use | ever | never | 35 | 0.000 | 55.4 | **0.93 (0.88, 0.99)** |
| Oral contraceptive use | ever | never | 23 | 0.014 | 43.5 | 1.02 (0.98, 1.07) |
| OC use duration (years) | ≥2 to ≥12 | 0 to <2 | 12 | 0.012 | 54.5 | 0.99 (0.87, 1.14) |
| Parity | **≥3 to ≥7** | **0 to 2** | **23** | **<0.001** | **76.0** | **0.82 (0.70, 0.95)** |
| Number of pregnancy | ≥ 4 to ≥7 | 0 to 2 | 8 | 0.014 | 60.1 | 0.84 (0.68, 1.04) |
| Age at first birth | **≥25 to ≥31** | **Nulliparous or <25** | **16** | 0.082 | **35.1** | **0.91 (0.83, 0.98)** |
| Reproductive period (years) | ≥36 to ≥41 | ≤30 to <33 | 8 | 0.048 | 50.6 | 0.95 (0.78, 1.17) |
| Breastfeeding | ever | never | 4 | 0.237 | 29.2 | 0.96 (0.84, 1.10) |
| Miscarriage | ever | never | 4 | 0.063 | 58.9 | 1.20 (0.93, 1.56) |
| Tubal sterilization use | ever | never | 3 | 0.035 | 70.1 | 1.00 (0.77, 1.29) |
| Intrauterine device use | ever | never | 4 | 0.097 | 52.5 | 0.83 (0.66, 1.04) |

^†^Heterogeneity P-value

^‡^Adjusted odds ratio (OR) and 95% confidence interval (CI). Highest non-reference category as compared to the lowest reference group.

**Reference：**

1. Vohra SN, Sapkota A, Lee MT, Pun CB, Thakur B, Siwakoti B, et al. Reproductive and Hormonal Factors in Relation to Lung Cancer Among Nepali Women. Front Oncol. 2019;9:311.

2. Jin K, Wu M, Zhou JY, Yang J, Han RQ, Jin ZY, et al. Tobacco Smoking Modifies the Association between Hormonal Factors and Lung Cancer Occurrence among Post-Menopausal Chinese Women. Transl Oncol. 2019;12(6):819-27.

3. Titan AL, He H, Lui N, Liou D, Berry M, Shrager J, et al. The Influence of Hormone Replacement Therapy on Lung Cancer Incidence and Mortality. The Journal of Thoracic and Cardiovascular Surgery. 2019.

4. Iversen L, Sivasubramaniam S, Lee AJ, Fielding S, Hannaford PC. Lifetime cancer risk and combined oral contraceptives: the Royal College of General Practitioners' Oral Contraception Study. Am J Obstet Gynecol. 2017;216(6):580 e1- e9.

5. He F, Xie JX, Liu CL, Xiong WM, Xu QP, Liu ZQ, et al. The relationship of lung cancer with menstrual and reproductive factors may be influenced by passive smoking, cooking oil fumes, and tea intake: A case-control study in Chinese women. Medicine (Baltimore). 2017;96(46):e8816.

6. Ben Khedher S, Neri M, Papadopoulos A, Christiani DC, Diao N, Harris CC, et al. Menstrual and reproductive factors and lung cancer risk: A pooled analysis from the international lung cancer consortium. Int J Cancer. 2017;141(2):309-23.

7. Patel MI, Wang A, Kapphahn K, Desai M, Chlebowski RT, Simon MS, et al. Racial and Ethnic Variations in Lung Cancer Incidence and Mortality: Results From the Women's Health Initiative. J Clin Oncol. 2016;34(4):360-8.

8. Schwartz AG, Ray RM, Cote ML, Abrams J, Sokol RJ, Hendrix SL, et al. Hormone Use, Reproductive History, and Risk of Lung Cancer: The Women's Health Initiative Studies. J Thorac Oncol. 2015;10(7):1004-13.

9. Tan HS, Tan MH, Chow KY, Chay WY, Lim WY. Reproductive factors and lung cancer risk among women in the Singapore Breast Cancer Screening Project. Lung Cancer. 2015;90(3):499-508.

10. Gallagher LG, Rosenblatt KA, Ray RM, Li W, Gao DL, Applebaum KM, et al. Reproductive factors and risk of lung cancer in female textile workers in Shanghai, China. Cancer Causes Control. 2013;24(7):1305-14.

11. Pesatori AC, Carugno M, Consonni D, Caporaso NE, Wacholder S, Tucker M, et al. Reproductive and hormonal factors and the risk of lung cancer: the EAGLE study. Int J Cancer. 2013;132(11):2630-9.

12. Lo YL, Hsiao CF, Chang GC, Tsai YH, Huang MS, Su WC, et al. Risk factors for primary lung cancer among never smokers by gender in a matched case-control study. Cancer Causes Control. 2013;24(3):567-76.

13. Lim WY, Chen Y, Chuah KL, Eng P, Leong SS, Lim E, et al. Female reproductive factors, gene polymorphisms in the estrogen metabolism pathway, and risk of lung cancer in Chinese women. Am J Epidemiol. 2012;175(6):492-503.

14. Brinton LA, Schwartz L, Spitz MR, Park Y, Hollenbeck AR, Gierach GL. Unopposed estrogen and estrogen plus progestin menopausal hormone therapy and lung cancer risk in the NIH-AARP Diet and Health Study Cohort. Cancer Causes Control. 2012;23(3):487-96.

15. Lin Y, Cai L. Environmental and dietary factors and lung cancer risk among Chinese women: a case-control study in southeast China. Nutr Cancer. 2012;64(4):508-14.

16. Meinhold CL, Berrington de Gonzalez A, Bowman ED, Brenner AV, Jones RT, Lacey JV, Jr., et al. Reproductive and hormonal factors and the risk of nonsmall cell lung cancer. Int J Cancer. 2011;128(6):1404-13.

17. Brinton LA, Gierach GL, Andaya A, Park Y, Schatzkin A, Hollenbeck AR, et al. Reproductive and hormonal factors and lung cancer risk in the NIH-AARP Diet and Health Study cohort. Cancer Epidemiol Biomarkers Prev. 2011;20(5):900-11.

18. Clague J, Reynolds P, Sullivan-Halley J, Ma H, Lacey JV, Jr., Henderson KD, et al. Menopausal hormone therapy does not influence lung cancer risk: results from the California Teachers Study. Cancer Epidemiol Biomarkers Prev. 2011;20(3):560-4.

19. Paulus JK, Asomaning K, Kraft P, Johnson BE, Lin X, Christiani DC. Parity and risk of lung cancer in women. Am J Epidemiol. 2010;171(5):557-63.

20. Baik CS, Strauss GM, Speizer FE, Feskanich D. Reproductive factors, hormone use, and risk for lung cancer in postmenopausal women, the Nurses' Health Study. Cancer Epidemiol Biomarkers Prev. 2010;19(10):2525-33.

21. Chlebowski RT, Anderson GL, Manson JE, Schwartz AG, Wakelee H, Gass M, et al. Lung cancer among postmenopausal women treated with estrogen alone in the women's health initiative randomized trial. J Natl Cancer Inst. 2010;102(18):1413-21.

22. Slatore CG, Chien JW, Au DH, Satia JA, White E. Lung cancer and hormone replacement therapy: association in the vitamins and lifestyle study. J Clin Oncol. 2010;28(9):1540-6.

23. Brenner DR, Hung RJ, Tsao MS, Shepherd FA, Johnston MR, Narod S, et al. Lung cancer risk in never-smokers: a population-based case-control study of epidemiologic risk factors. Bmc Cancer. 2010;10.

24. Heck JE, Andrew AS, Onega T, Rigas JR, Jackson BP, Karagas MR, et al. Lung Cancer in a US Population with Low to Moderate Arsenic Exposure. Environ Health Persp. 2009;117(11):1718-23.

25. Seow A, Koh WP, Wang R, Lee HP, Yu MC. Reproductive variables, soy intake, and lung cancer risk among nonsmoking women in the Singapore Chinese Health Study. Cancer Epidemiol Biomarkers Prev. 2009;18(3):821-7.

26. Dorjgochoo T, Shu XO, Li HL, Qian HZ, Yang G, Cai H, et al. Use of oral contraceptives, intrauterine devices and tubal sterilization and cancer risk in a large prospective study, from 1996 to 2006. Int J Cancer. 2009;124(10):2442-9.

27. Chlebowski RT, Schwartz AG, Wakelee H, Anderson GL, Stefanick ML, Manson JE, et al. Oestrogen plus progestin and lung cancer in postmenopausal women (Women's Health Initiative trial): a post-hoc analysis of a randomised controlled trial. The Lancet. 2009;374(9697):1243-51.

28. Smith JR, Barrett-Connor E, Kritz-Silverstein D, Wingard DL, Al-Delaimy WK. Hormone use and lung cancer incidence: the Rancho Bernardo cohort study. Menopause. 2009;16(5):1044-8.

29. Koushik A, Parent ME, Siemiatycki J. Characteristics of menstruation and pregnancy and the risk of lung cancer in women. Int J Cancer. 2009;125(10):2428-33.

30. Rosenblatt KA, Gao DL, Ray RM, Nelson ZC, Wernli KJ, Li W, et al. Oral contraceptives and the risk of all cancers combined and site-specific cancers in Shanghai. Cancer Causes Control. 2009;20(1):27-34.

31. Weiss JM, Lacey JV, Jr., Shu XO, Ji BT, Hou L, Yang G, et al. Menstrual and reproductive factors in association with lung cancer in female lifetime nonsmokers. Am J Epidemiol. 2008;168(11):1319-25.

32. Ramnath N, Menezes RJ, Loewen G, Dua P, Eid F, Alkhaddo J, et al. Hormone replacement therapy as a risk factor for non-small cell lung cancer: results of a case-control study. Oncology. 2007;73(5-6):305-10.

33. Rodriguez C, Spencer Feigelson H, Deka A, Patel AV, Jacobs EJ, Thun MJ, et al. Postmenopausal hormone therapy and lung cancer risk in the cancer prevention study II nutrition cohort. Cancer Epidemiol Biomarkers Prev. 2008;17(3):655-60.

34. Corrao G, Zambon A, Conti V, Nicotra F, La Vecchia C, Fornari C, et al. Menopause hormone replacement therapy and cancer risk: an Italian record linkage investigation. Ann Oncol. 2008;19(1):150-5.

35. Mahabir S, Spitz MR, Barrera SL, Dong YQ, Eastham C, Forman MR. Dietary boron and hormone replacement therapy as risk factors for lung cancer in women. Am J Epidemiol. 2008;167(9):1070-80.

36. Schwartz AG, Wenzlaff AS, Prysak GM, Murphy V, Cote ML, Brooks SC, et al. Reproductive factors, hormone use, estrogen receptor expression and risk of non small-cell lung cancer in women. J Clin Oncol. 2007;25(36):5785-92.

37. Kabat GC, Miller AB, Rohan TE. Reproductive and hormonal factors and risk of lung cancer in women: a prospective cohort study. Int J Cancer. 2007;120(10):2214-20.

38. Matsuo K, Ito H, Yatabe Y, Hiraki A, Hirose K, Wakai K, et al. Risk factors differ for non-small-cell lung cancers with and without EGFR mutation: assessment of smoking and sex by a case-control study in Japanese. Cancer Sci. 2007;98(1):96-101.

39. Chen KY, Hsiao CF, Chang GC, Tsai YH, Su WC, Perng RP, et al. Hormone replacement therapy and lung cancer risk in Chinese. Cancer. 2007;110(8):1768-75.

40. Elliott AM, Hannaford PC. Use of exogenous hormones by women and lung cancer: evidence from the Royal College of General Practitioners' Oral Contraception Study. Contraception. 2006;73(4):331-5.

41. Gorlova OY, Zhang Y, Schabath MB, Lei L, Zhang Q, Amos CI, et al. Never smokers and lung cancer risk: a case-control study of epidemiological factors. Int J Cancer. 2006;118(7):1798-804.

42. Liu Y, Inoue M, Sobue T, Tsugane S. Reproductive factors, hormone use and the risk of lung cancer among middle-aged never-smoking Japanese women: a large-scale population-based cohort study. Int J Cancer. 2005;117(4):662-6.

43. Schabath MB, Wu XF, Vassilopoulou-Sellin R, Vaporciyan AA, Spitz MR. Hormone replacement therapy and lung cancer risk. A case-control analysis. Cancer Epidem Biomar. 2003;12(11):1294s-s.

44. Kreuzer M, Gerken M, Heinrich J, Kreienbrock L, Wichmann HE. Hormonal factors and risk of lung cancer among women? International Journal of Epidemiology. 2003;32(2):263-71.

45. Olsson H, Bladstrom A, Ingvar C. Are smoking-associated cancers prevented or postponed in women using hormone replacement therapy? Obstetrics and Gynecology. 2003;102(3):565-70.

46. Zatloukal P, Kubik A, Pauk N, Tomasek L, Petruzelka L. Adenocarcinoma of the lung among women: risk associated with smoking, prior lung disease, diet and menstrual and pregnancy history. Lung Cancer. 2003;41(3):283-93.

47. Brenner AV, Wang ZY, Kleinerman RA, Lei SJ, Metayer C, Wang WL, et al. Menstrual and reproductive factors and risk of lung cancer among Chinese women, Eastern Gansu Province, 1994-1998. Journal of Epidemiology. 2003;13(1):22-8.

48. Hulley S, Furberg C, Barrett-Connor E, Cauley J, Grady D, Haskell W, et al. Noncardiovascular disease outcomes during 6.8 years of hormone therapy - Heart and Estrogen/progestin Replacement Study follow-up (HERS II). Jama-J Am Med Assoc. 2002;288(1):58-66.

49. Seow A, Poh WT, Teh M, Eng P, Wang YT, Tan WC, et al. Diet, reproductive factors and lung cancer risk among Chinese women in Singapore: evidence for a protective effect of soy in nonsmokers. Int J Cancer. 2002;97(3):365-71.

50. Kubik AK, Zatloukal P, Tomasek L, Petruzelka L. Lung cancer risk among Czech women: a case-control study. Prev Med. 2002;34(4):436-44.

51. Blackman JA, Coogan PF, Rosenberg L, Strom BL, Zauber AG, Palmer JR, et al. Estrogen replacement therapy and risk of lung cancer. Pharmacoepidemiology and Drug Safety. 2002;11(7):561-7.

52. Rossouw JE, Anderson GL, Prentice RL, LaCroix AZ, Kooperberg C, Stefanick ML, et al. Risks and benefits of estrogen plus progestin in healthy postmenopausal women: principal results From the Women's Health Initiative randomized controlled trial. JAMA. 2002;288(3):321-33.

53. Pukkala E, Tulenheimo-Silfvast A, Leminen A. Incidence of cancer among women using long versus monthly cycle hormonal replacement therapy, Finland 1994-1997. Cancer Cause Control. 2001;12(2):111-5.

54. Zhou BS, Wang TJ, Guan P, Wu JM. Indoor air pollution and pulmonary adenocarcinoma among females: A case-control study in Shenyang, China. Oncology Reports. 2000;7(6):1253-9.

55. Persson I, Yuen J, Bergkvist L, Schairer C. Cancer incidence and mortality in women receiving estrogen and estrogen-progestin replacement therapy - Long-term follow-up of a Swedish cohort. International Journal of Cancer. 1996;67(3):327-32.

56. Taioli E, Wynder EL. Endocrine Factors and Adenocarcinoma of the Lung in Women. J Natl Cancer I. 1994;86(11):869-70.

57. Wuwilliams AH, Dai XD, Blot W, Xu ZY, Sun XW, Xiao HP, et al. Lung-Cancer among Women in North-East China. Brit J Cancer. 1990;62(6):982-7.

58. Adami HO, Persson I, Hoover R, Schairer C, Bergkvist L. Risk of Cancer in Women Receiving Hormone Replacement Therapy. International Journal of Cancer. 1989;44(5):833-9.

59. Wu AH, Yu MC, Thomas DC, Pike MC, Henderson BE. Personal and Family History of Lung-Disease as Risk-Factors for Adenocarcinoma of the Lung. Cancer Research. 1988;48(24):7279-84.

60. Gao YT, Blot WJ, Zheng W, Ershow AG, Cheng WH, Levin LI, et al. Lung-Cancer among Chinese-Women. International Journal of Cancer. 1987;40(5):604-9.

61. Xiang Yea. 2003 A population-based case-control study of lung cancer between young and older nonsmoking women in urban Shanghai, P. R. China. (in Chinese). Tumor. 2003;23(6):452-7

62. Chen Wea. A cohort study on risk factors of Iung cancer among nonsmoking women in urban Shanghai. (in Chinese). Fudan University. 2009.

63. Y L, X C, M H. A case-control study of risk factors for female lung cancer. (in Chinese). J Fujian Med Univ. 2010;44(4):239-43.

64. W Z, YT G, L S. A study on the association between lung cancer and menstrual and reproductive history. (in Chinese). Tumor. 1988;8(3):150-3.

65. Yin Zea. A case-control study on relationship between lung cancer in non-smoking women and menstrual and reproductive factors. (in Chinese). Chin J Public Health. 2005;21(12):1456-7.

66. Li Xea. Risk status analysis of female lung cancer. (in Chinese). J Jinan University. 1995;16(2):18-22.
