# Supplementary material for: Reproductive factors and lung cancer risk: a comprehensive systematic review and meta-analysis
Source: BMC Public Health. 2020 Sep 25;20:1458. doi: 10.1186/s12889-020-09530-7 (PMC7519481; doi:10.1186/s12889-020-09530-7)
Supplement: Supplementary file 2 — Additional file 2: Supplementary Figs. S1–13. Forest plots for lung cancer risks in women with reproductive variables [file 12889_2020_9530_MOESM2_ESM.docx]

| 1. **Hormone use** | 1. **Oral contraceptive use** | 1. **Parity** | 1. **Age at menopause** |
| --- | --- | --- | --- |
| 1. **Age at menarche** | 1. **Age at first birth** | 1. **OC use duration (years)** | 1. **Menopause type (reason)** |
| 1. **Number of pregnancy** | 1. ** Reproductive period (years)** | 1.  **Menstrual cycle length** | 1. **Breastfeeding** |
| 1. **Menopausal status** | **14) Ovariectomy** | **15) Miscarriage** | **16) Length of menstrual flow** |
| **17) Tubal sterilization use** | **18) Intrauterine device use** | **19) Hysterectomy** | **20) Uterine and ovarian all cut** |

**Supplementary Figure S1.** **Forest plots for overall lung cancer risks in women with reproductive variables.**

| ****a)** | **b)** | **c) ** |
| --- | --- | --- |
| **d)** | **e)** | **f)** |

**Supplementary Figure S2. Forest plots for the association between parity and lung cancer risks in women (≥3 children vs. 0-2 children ).** a) Overall lung cancer risks stratified by study designs; b) Overall lung cancer risks stratified by ethnicity; c) Non-small cell lung cancer risks stratified by study designs; d) Non-small cell lung cancer risks stratified by ethnicity; e) adenocarcinoma risks stratified by study designs; f) adenocarcinoma risks stratified by ethnicity.

| **a)** | **b)** | **c)** |
| --- | --- | --- |
| **d)** | **e)** | **f)** |

**Supplementary Figure S3. Forest plots for the association between parity and lung cancer risks in Asian and Caucasian women stratified by study designs (≥3 children vs. 0-2 children).** a) Asian women with lung cancer; b) Caucasian women with lung cancer; c) Asian women with non-small cell lung cancer; d) Caucasian women with non-small cell lung cancer; e) Asian women with adenocarcinoma; f) Caucasian women with adenocarcinoma.

| **a)** | **b)** |
| --- | --- |
| **c)** | **d)** |

**Supplementary Figure S4. Forest plots for the association between parity and lung cancer risks in women (****≥1 child vs. nulliparous).** a) Overall lung cancer risks stratified by study designs; b) overall lung cancer risks stratified by ethnicity; c) adenocarcinoma risks stratified by study designs; d) adenocarcinoma risks stratified by ethnicity.

| **a)** | **b)** |
| --- | --- |
| **c)** | **d)** |

**Supplementary Figure S5.** **Forest plots for the association between parity and lung cancer risks in Asian and Caucasian women stratified by study designs (≥1 child vs. nulliparous).** a) Asian women with lung cancer; b) Asian women with adenocarcinoma; c) Caucasian women with lung cancer; d) Caucasian women with adenocarcinoma.

| **a)** | **b)** |
| --- | --- |
| **c)** | **d)** |

**Supplementary Figure S6. Forest plots for the association between parity and lung cancer risks in never-smoked vs. ever smoked women stratified by study designs and ethnicity (****≥3 children vs. 0-2 children).** a) never smokers stratified by study design; b) never smokers stratified by ethnicity; c) ever smokers stratified by study design; d) ever smokers stratified by ethnicity;

| **a)** | **b)** |
| --- | --- |
| **c)** | **d)** |

**Supplementary Figure S7. Forest plots for the association between parity and lung cancer risks in never-smoked vs. ever smoked women stratified by study designs and ethnicity (****≥1 child vs. Nulliparous).** a) Never smokers stratified by study design; b) never smokers stratified by ethnicity; c) ever smokers stratified by study design; d) ever smokers stratified by ethnicity.

| **** |
| --- |

**Supplementary Figure S8. Forest plots for lung cancer risks in never-smoked vs. ever smoked women with reproductive variables of parity (≥3 children vs. 0-2 children).**

| **a)** | **b)** |
| --- | --- |
| **c)** | **d)** |

**Supplementary Figure S9. Forest plots for the association between age at first birth and lung cancer risks in women (≥25 vs. Nulliparous / <25).** a) Overall lung cancer risks stratified by study designs; b) overall lung cancer risks stratified by ethnicity; c) adenocarcinoma risks stratified by study designs; d) adenocarcinoma risks stratified by ethnicity.

| **a)** | **b)** |
| --- | --- |
| **c)** | **d)** |

**Supplementary Figure S10. Forest plots for the association between menopause reason and lung cancer risks in women (non-natural menopause versus natural menopause).** a) Overall lung cancer risks stratified by study designs; b) overall lung cancer risks stratified by ethnicity; c) adenocarcinoma risks stratified by study designs; d) adenocarcinoma risks stratified by ethnicity.

| **a)** | **b)** |
| --- | --- |
| **cc)** | **d)** |

**Supplementary Figure S11. Forest plots for the association between menstrual cycle length and lung cancer risks in women (>30 days vs. <27 to ≤ 30 days).**  a) Overall lung cancer risks stratified by study designs; b) overall lung cancer risks stratified by ethnicity; c) adenocarcinoma risks stratified by study designs; d) adenocarcinoma risks stratified by ethnicity.

| **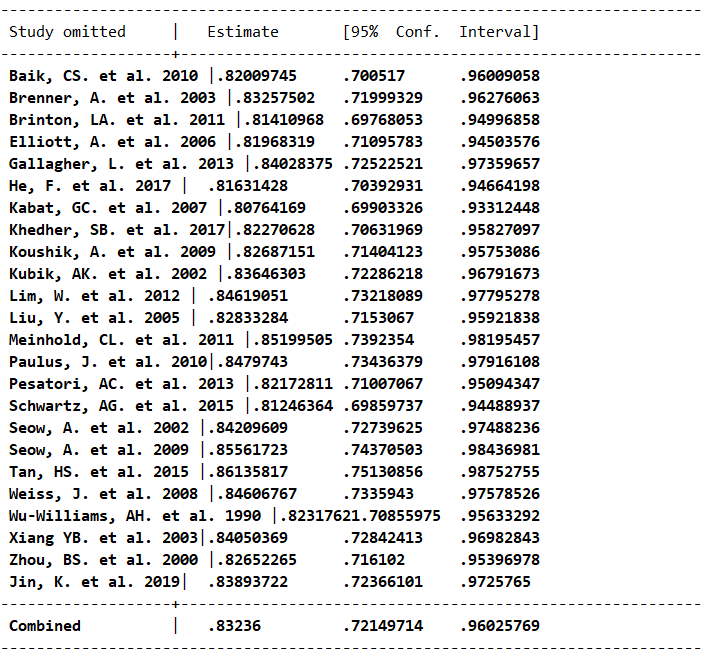a)** |
| --- |
| **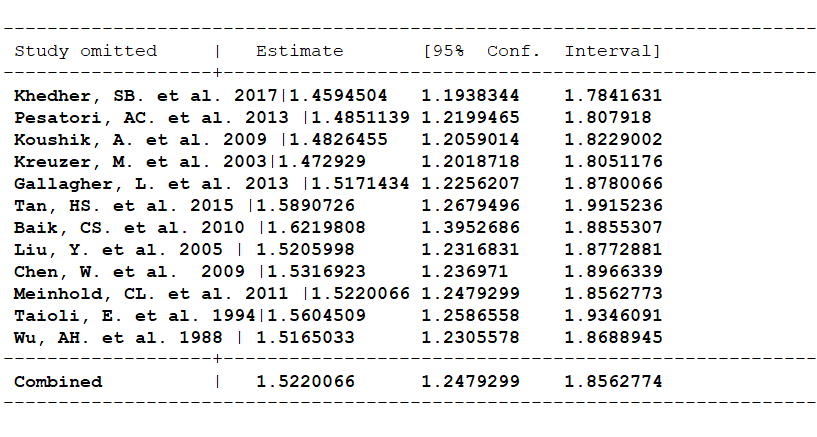****b)** |

**Supplementary Figure S12. Sensitivity analysis for:** a) parity; b) menopause type**;**

| **a)** |
| --- |
| ******b)** |

**Supplementary Figure S13. Funnel plots and funnel plots with trim and fill for:** a) parity; b) menopause type.
